# MiR-27a Functions as a Tumor Suppressor in Acute Leukemia by Regulating 14-3-3θ

**DOI:** 10.1371/journal.pone.0050895

**Published:** 2012-12-07

**Authors:** Kara A. Scheibner, Brianne Teaboldt, Mary Claire Hauer, Xiaochun Chen, Srujana Cherukuri, Yin Guo, Shannon M. Kelley, Zhenqiu Liu, Maria R. Baer, Shelly Heimfeld, Curt I. Civin

**Affiliations:** 1 Center for Stem Cell Biology and Regenerative Medicine, University of Maryland School of Medicine, Baltimore, Maryland, United States of America; 2 Department of Pediatrics, University of Maryland School of Medicine, Baltimore, Maryland, United States of America; 3 Department of Epidemiology and Public Health, University of Maryland School of Medicine, Baltimore, Maryland, United States of America; 4 Department of Medicine, University of Maryland School of Medicine, Baltimore, Maryland, United States of America; 5 Clinical Research Division, Fred Hutchinson Cancer Research Center, Seattle, Washington, United States of America; Virginia Commonwealth University, United States of America

## Abstract

MicroRNAs (miRs) play major roles in normal hematopoietic differentiation and hematopoietic malignancies. In this work, we report that miR-27a, and its coordinately expressed cluster (miR-23a∼miR-27a∼miR-24-2), was down-regulated in acute leukemia cell lines and primary samples compared to hematopoietic stem-progenitor cells (HSPCs). Decreased miR-23a cluster expression in some acute leukemia cell lines was mediated by c-MYC. Replacement of miR-27a in acute leukemia cell lines inhibited cell growth due, at least in part, to increased cellular apoptosis. We identified a member of the anti-apoptotic 14-3-3 family of proteins, which support cell survival by interacting with and negatively regulating pro-apoptotic proteins such as Bax and Bad, as a target of miR-27a. Specifically, miR-27a regulated 14-3-3θ at both the mRNA and protein levels. These data indicate that miR-27a contributes a tumor suppressor-like activity in acute leukemia cells via regulation of apoptosis, and that miR-27a and 14-3-3θ may be potential therapeutic targets.

## Introduction

MicroRNAs (miRs) are ∼22 nt, non-coding RNA molecules that play roles in most cellular processes, including apoptosis [1]. Dysregulation of miRs has been implicated in many disease states, prominently including cancer, where there are significant differences in miR expression profiles of cancer cells versus normal cells from the tissues of origin [2]. MiRs negatively regulate gene expression by binding to partially complementary sites (mediating mainly translational inhibition or mRNA destabilization) or, less frequently, fully complementary sites (mediating mainly mRNA degradation) [3], [4]. These target sites can be located in the 3′UTR, the coding region (CDS), and/or the 5′UTR of target gene mRNA [3], [4]. In cancer, a miR can function as either a tumor suppressor gene (e.g., miR-15a∼16-1, let-7 family) or an oncogene (e.g., miR-155, miR-17∼92) [5].

We and others have found that miRs play major regulatory roles in normal hematopoietic differentiation, evidenced by the discovery of a small set of hematopoietic stem-progenitor cell (HSPC)-expressed miRs (HE-miRs) which post-transcriptionally regulate specific mRNAs involved in hematopoiesis [6]–[9]. MiR-23a and miR-24 were identified as enriched in CD34+ HSPCs [6], and miR-24 has a well-defined role in as a regulator of normal erythropoiesis via targeting of human activin receptor type1, ALK4 [10]. Additionally, miR-23a and miR-24 both act as tumor suppressors and are down-regulated in multiple cancers. MiR-23a and miR-24-2 are coordinately transcribed and generally expressed at similar levels with a co-localized third miR, miR-27a. This “miR-23a cluster” (miR-23a∼miR-27a∼miR-24-2) is located on human chromosome 19p13.2, [11], (Figure S1) and is expressed from its own upstream promoter, located in the −600 to +36 bp region, which includes a GC-rich region and a transcription start site (0 to 124 bp) [12], [13]. There is also a “miR-23b cluster” (miR-23b∼miR-27b∼miR-24-1), which is located on chromosome 9 in C9orf3. These 2 paralogous clusters are under different regulatory controls and hence are differentially expressed in cells [13], yet both the miR-23a and miR-23b clusters are under direct negative regulation by c-MYC [14]. Expression of all 3 miR-23a cluster members is down-regulated in acute promyelocytic leukemia (APML), colorectal cancer, oral squamous cell carcinoma, and prostate cancer, though there are rare cases where miR-23a cluster member expression is not correlated [13].

While the expression and effects of miR-23a and miR-24 are well-described in cancer and hematopoiesis, less is known regarding miR-27a. MiR-27a, in conjunction with RUNX1, regulates normal megakaryocytic differentiation [9]. MiR-27a is up-regulated in estrogen receptor-positive breast cancers (regulating ZBTB10) and gastric cancers [13], and down-regulated in malignant melanoma and the aforementioned cancers where cluster expression is decreased [13]. In this study, we determined the expression of miR-27a in CD34+ HSPCs and its expression and effects in human acute leukemias. Our data indicate that miR-27a acts as an acute leukemia suppressor.

## Experimental Procedures

### Cells and primary samples

Acute leukemia cell lines were acquired from American Type Culture Collection (ATCC, Manassas, VA) and Deutsche Sammlung von Mikroorganismen und Zellkulturen GmbH (DSMZ, Braunschweig, Germany), and cultured as recommended. Cell lines used experimentally were K562, TF1, HL60, REH, KOPN8, SUPB15, Molt16, Karpas45, and HEK293T. Additional cell lines used for profiling only were Kasumi1, KG1a, ML2, M07e, and U937 (AML); Kasumi2, MHH CALL3, MHH CALL4, MUTZ5, NALM6, and RCH ACV (pre-B-ALL); and CCRFCEM, Jurkat, and MOLT3 (T-ALL). Human CD34^+^ peripheral blood mononuclear cells (PBMCs) from normal adult donors were obtained from the National Heart, Lung, and Blood Institute Program of Excellence in Gene Therapy, Hematopoietic Cell Processing Core (Fred Hutchison Cancer Center) [6]. Primary human acute leukemia samples were isolated from spleens of engrafted immunodeficient mice [15] or obtained as frozen primary samples (from the University of Maryland or Johns Hopkins University School of Medicine cell banks); each patient sample was assigned a unique identifying number.

### Isolation of RNA and measurement of miR levels

RNA was isolated using the miRNeasy kit (Qiagen, Valencia, CA) according to the manufacturer's protocol. Levels of mature miRs were measured in a 7900 Real-Time PCR System (Applied Biosystems, Inc, Carlsbad, CA) using TaqMan qRT-PCR (Applied Biosystems); U18 was chosen as an endogenous control based on its consistent expression across acute leukemia cell lines and primary samples. The expression of each miR in each sample was normalized to the average level of that miR in CD34+ HSPCs and expressed as fold difference (2^−ΔΔCt^±1 standard error of the mean, SEM). MiR levels were also measured by microarray analysis; RNA was isolated as above and 500 ng RNA per sample was hybridized to each GeneChip miRNA 2.0 Array (Affymetrix, Santa Clara, CA) which contained 46,228 probes for the miRs of 71 organisms. mRNA microarray expression analysis was performed by hybridizing 100 ng RNA to each GeneChip Human Gene 1.0 ST Array (Affymetrix), containing 764,885 probes representing 28,869 annotated genes. cRNA synthesis, labeling, hybridization to arrays, washing, staining, and signal amplification were performed according to manufacturer's instructions in the core facilities of the University of Maryland School of Medicine. Data analysis was performed using GeneSpring GX (Agilent Technologies, Palo Alto, CA); both miR and mRNA normalizations used the robust multi-chip average (RMA) method, generating log 2 transformed intensity values. Correlations of miR expression levels were determined by Pearson correlation; r>0.300 and p<0.05 were considered significant.

### Determination of the effect of c-MYC on miR-23a cluster expression

P493B cells were a generous gift from Chi van Dang (University of Pennsylvania) [14]. Cells were treated with doxycycline (Dox) for 48 hr, harvested, and total RNA isolated as above. MiR-23a cluster member expression levels were measured via qRT-PCR and normalized to levels in untreated cells (2^−ΔΔCt^±SEM). Levels of miR-27a and c-MYC mRNA expression in acute leukemia cell lines and primary samples were measured via microarray and normalized as above. Correlations between cellular miR-27a and c-MYC levels were determined by Pearson correlation (r<−0.3000). Cell lines were treated with 25 µM or 50 µM of the pharmacologic c-MYC inhibitor 10058-F4 (Calbiochem, Gibbstown, NJ) or with DMSO (vehicle) for 48 hr. Expression levels of miR-23a and miR-27a in total cellular RNA were assessed by TaqMan qRT-PCR, as above. c-MYC was knocked down in K562, MOLT16, and Karpas45 cells via transfection with 50 nM or 100 nM of the siGENOME SMART pool c-MYC siRNA (Dharmacon, Lafayette, CO) or 100 nM of control siRNA. After 72 hr, total RNA was harvested, and miR-23a and miR-27a expression levels analyzed via TaqMan qRT-PCR. Protein lysates were isolated to demonstrate down-regulation of c-MYC protein via Western blot (c-MYC antibody S-40, Santa Cruz Biotechnology (SCB), Santa Cruz, CA) with GAPDH (S-137179, SCB) as loading control.

### Enforced expression of miR-27a

Expression of miR-27a was enforced using a dual promoter lentiviral vector (FUGW backbone) as described [16]. Cells were transduced with control virus (FUGW) or miR-27a-expressing virus (FUGW/miR-27a) at a multiplicity of infection (MOI) = 5 for 72 hr. Cells were plated in methylcellulose-containing colony-forming assay medium (StemCell Technologies, Vancouver, BC) in triplicate at 400 cells/ml/plate, or in suspension (1 cell/well) in a microtiter plate in a limiting dilution strategy. GFP+ colonies were selected via fluorescence microscopy and then expanded in suspension cultures for 7–10 days, and analyzed for GFP via fluorescence activated cell sorting (FACS) and miR-27a (qRT-PCR) expression.

### Determination of cell viability and apoptosis

K562 and REH cell viability (by FACS side scatter vs. forward scatter) of FUGW- and FUGW/miR-27a-transduced colonies was measured weekly after isolation from colonies in methylcellulose cultures. Cell viability was also measured via propidium iodide (PI) staining; FUGW/miR-27a- or FUGW-transduced cells (10^6^ cells/ml media), derived from a clonal population and subsequently expanded for 7 days, were stained with 2 µl PI (500 µg/ml) and FACS-analyzed. Percent apoptosis in cells transduced and isolated as above was determined via correlated AnnexinV and 7-Aminoactinomycin D (7AAD) staining; ∼10^6^ cells were stained with AnnexinV and 7AAD (BD Biosciences Franklin Lake, NJ, according to manufacturer's protocol) and FACS-analyzed. Percent apoptosis was taken as the percent AnnexinV^+^/7AAD^−^ signals and compared by the Student's t-test (n>3). Correlation between PI+ staining and GFP+ expression in Molt16 cells was determined by FACS analysis on cells isolated by limiting dilution. FACS was also used to determine the change in GFP expression over time in Molt16 cells.

### Tet-inducible miR-27a construct

pTRIPZ (Open Biosystems, Huntsville, AL), a Tet-On vector, was used to make K562 stable cell lines that express miR-27a inducibly. The DNA fragment encoding pre-miR27a was amplified from human CD34+ HSPC genomic DNA by PCR using the following primers: 5′TACCTCGAGCTGAGCTCTGCCAC CGAGGAT and 3′ TCAGAATTCGAGG CCAGGCAGCAGGATGGC. This PCR product was cloned into the pTRIPZ vector using XhoI and EcoRI sites. The construct (TG-27a) and control (TG) vector were packaged into lentivirus according to the manufacturer's protocol; stable cell lines were also made according to the manufacturer's protocol. The effect of Dox on cell growth 48 hrs after treatment was determined with the CellTiter 96® AQueous One Solution Cell Proliferation Assay (Promega, Madison, WI).

### Western Blots

K562 cells were transfected with 50 nM mature miR-23a, miR-27a, or miR-24, or with a combination of ≥2 miR mimics (Dharmacon, Lafayette, CO). Cells were harvested 48 hr after transfection and protein concentration determined by Bradford assay (Bio-Rad, Hercules, CA). 12.5 µg protein from each sample was loaded on a precast 4–12% MOPS gradient gel (Invitrogen), and stained with primary antibodies for 14-3-3θ (3B9, sc-59414), 14-3-3β (A-6, sc-25276), 14-3-3ζ (C-16, sc-1019), 14-3-3γ (6A1, sc-69955), or 14-3-3ε (8C3, sc-23957) according to manufacturer's recommendations (Santa Cruz Biotechnology, Santa Cruz, CA). Blots were imaged on a LAS-3000 image analyzer (Fuji, Tokyo, Japan) and quantified using Imagequant 4A.22 software (Fuji); γ-tubulin (Santa Cruz, C-20, sc-7396) was the loading normalization control.

### Luciferase assays

Predicted miR-27a and miR-24 binding sites from the 3′UTR or CDS of 14-3-3θ, 14-3-3β, or 14-3-3ζ, along with ∼50–100 surrounding bases from the genomic sequence on each side, were cloned into our luciferase (Luc) modified pcDNA3.1 plasmid, using EcoRI and XhoI as described [6] (Table S1). Because of difficulties cloning the miR-24 predicted binding sites in the CDS region, commercially synthesized oligonucleotides corresponding to the predicted binding regions flanked by 22 nt and with the pre-cut cloning sites for EcoRI and XhoI on either side (Integrated DNATechnologies, Coralville, IA) were obtained. The sequence for each strand was as follows: Strand 1: 5′- **AATTC**AGAAAGCCAAACTCGCTGAGCAGGCTGAGCGATATGA TGATATGGC TGCAGCCATGAAGGCAG T**C**-3′. Strand 2: 5′-**TCGAG**ACTGCCTT CATGGCTGCAG CCATATCATCATATCGCTCAGCCTGCTCAGCGAGTTTGGC TTTCT**G**-3′ (Underlined bold bases correspond to “pre-cut” enzyme cloning sites). The 2 strands were annealed together following the Invitrogen annealing protocol. The annealed duplex was ligated into the Luc reporter plasmid. Deletion mutants were made via PCR with the QuickChange mutation kit (Stratagene, La Jolla, CA) according to the manufacturer's protocol (Table S1). 500 ng of the Luc plasmid or the comparable deletion mutant, was co-transfected (using Lipofectamine 2000, Invitrogen), with or without artificial miR-23a, miR-27a, or miR-24 mimics (25 nM, Dharmacon), into HEK293T cells. A β-galactosidase (β-gal) plasmid (50 ng) was co-transfected for normalization [6]. Cells were lysed 24 hr after transfection, and Luc and β-gal activity measured as described [6]. Significant differences in normalized Luc activity in the presence of the miR vs. normalized Luc activity with plasmid alone were determined by Student's t-test (n≥3).

### 14-3-3θ open reading frame cloning and rescue experiments

The open reading frame (ORF) of 14-3-3θ was cloned into the pcDNA3.1 mammalian expression vector using XhoI and EcoRI restriction sites. The following primers were used: CATGGAATTCATGGAGAAGACTGAGCTGATCCAG (forward) and CGCTCGAGTTA GTTTTCAGCCCCTTCTGCCGC (reverse). FUGW/miR-27a- and FUGW-transduced K562 clones were transfected with 2 µg 14-3-3θ ORF or empty vector; cells were analyzed 48 hr and 72 hr post-transfection for %PI+ cells by FACS. Significant differences between 14-3-3θ ORF-transfected clones and control-transfected clones were determined by Student's t-test (n≥3).

## Results

### MiR-23a cluster expression levels are low in 90% of precursor-B ALL and T cell ALL cell lines and primary samples

**Figure 1:**
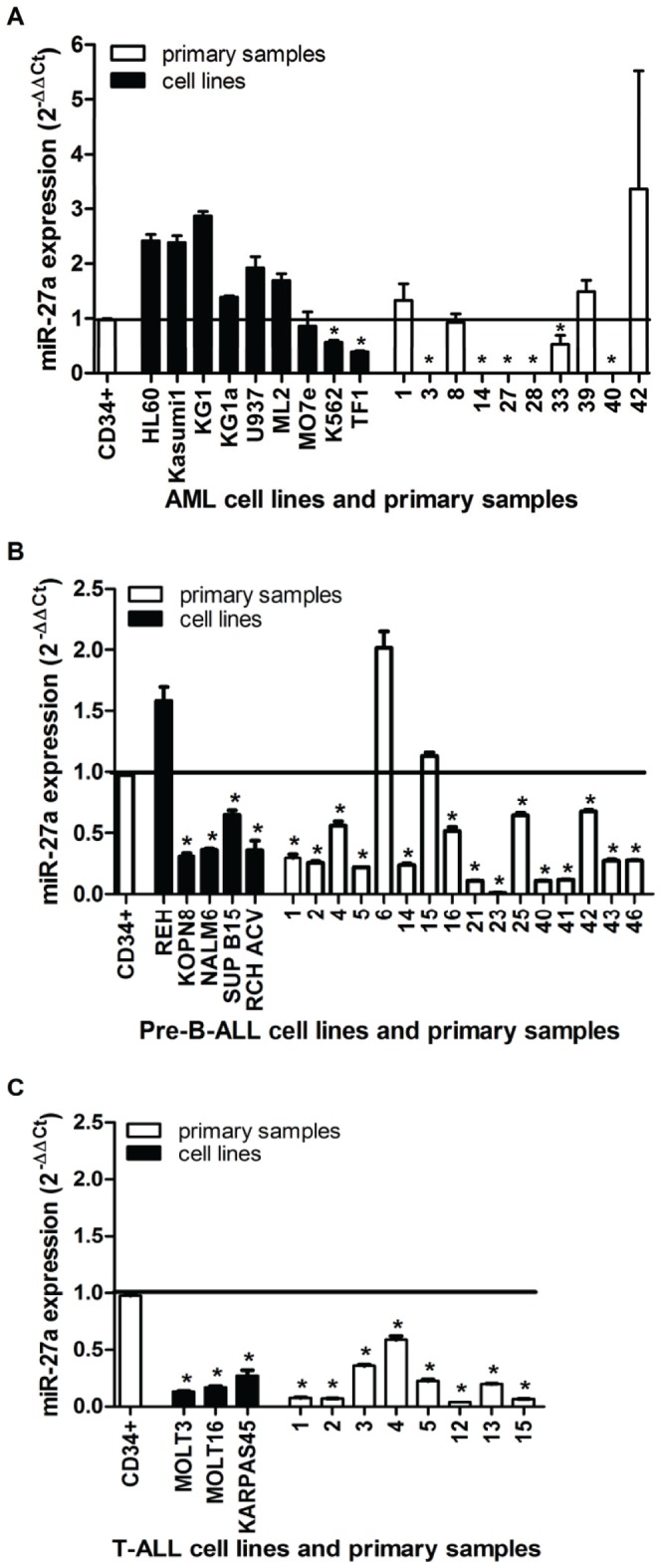
Expression levels of mature miR-27a in AML, pre-B-ALL, and T-ALL cell lines and primary samples. Total RNA was isolated from human AML (A), pre-B-ALL (B), and T-ALL (C) cell lines (black bars) and primary cases (primary patient cases identified with unique numbers, white bars). MiR-27a expression levels were normalized to mean levels in normal human CD34+ HSPCs (2^−ΔΔCt^ = 0.9756±0.022, black line). A Student's t-test was used to determine the significance of the difference in mean miR-27a expression (±SEM) between each leukemia samples and normal CD34+ HSPCs; significance is indicated as p<0.05 (*). n≥3 independent experiments in all cases except for the 10 primary AMLs which were analyzed in triplicate due to limited sample quantity.

**Figure 2:**
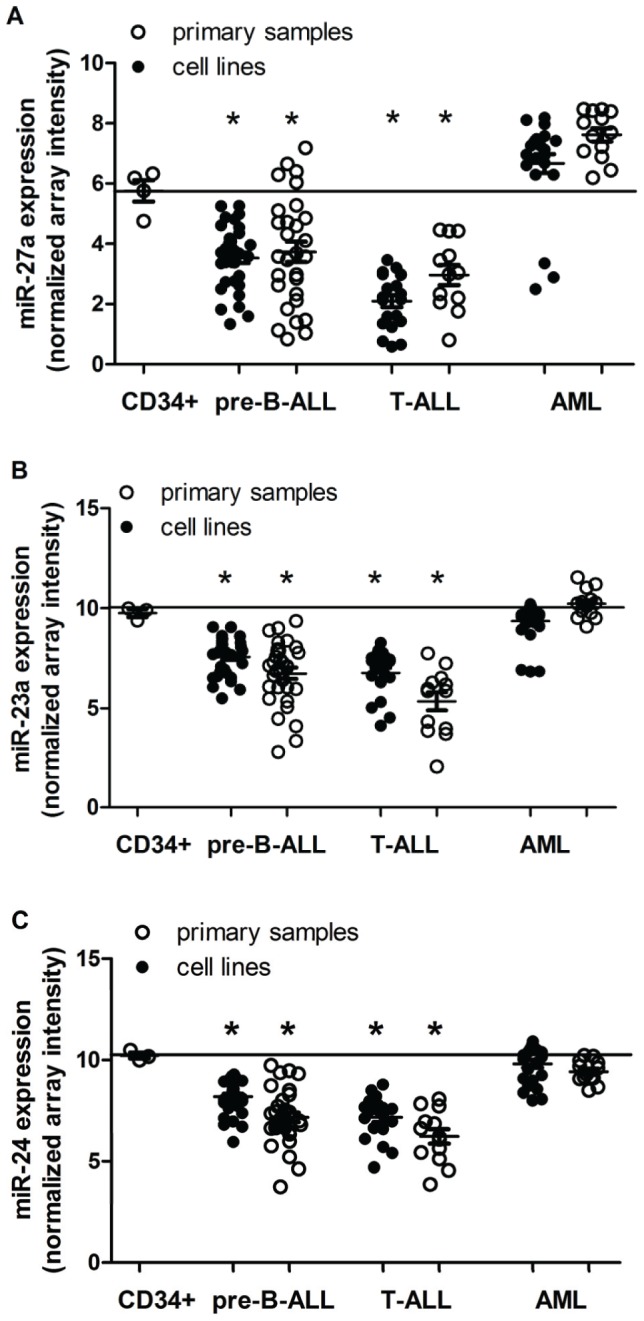
MiR-23a cluster expression in pre-B-ALL, T-ALL, and AML cell lines and primary samples. Expression of miR-27a (A), miR-23a (B), and miR-24 (C) was determined via microarray analysis in pre-B-ALL, T-ALL, and AML. (A) Average miR-27a expression (±SEM, p<0.05*) was compared to expression in normal CD34+ HSPCs (5.76±0.35, represented by black line) in pre-B-ALL cell lines (n = 36 [replicates of 9 cell lines]), pre-B-ALL primary samples (n = 16), T-ALL cell lines (n = 23 [replicates of 5 cell lines]), T-ALL primary samples (n = 13), AML cell lines (n = 24 [replicates of 7 cell lines]), and AML primary samples (n = 13). (B) Average miR-23a expression (±SEM, p<0.05*) was compared to normal CD34+ HSPCs (9.76±0.19, represented by black line). (C) Average miR-24 expression (±SEM, p<0.05*) was compared to expression in normal CD34+ HSPCs (10.23±0.14). n values for (B) and (C) were as in (A).

In normal human CD34+ HSPCs, miR-23a, miR-27a, and miR-24 were expressed at high levels (Ct<29 by qRT-PCR), similar to the U18 endogenous positive control (Figure S2A). However, in 86% of precursor B-cell acute lymphocytic leukemia (pre-B-ALL),100% of T-cell acute lymphocytic leukemia (T-ALL) cell lines and primary samples (18 of 21 and 11 of 11 samples respectively) and 42% of acute myeloid leukemia (AML) cell lines and primary samples (8 of 19 samples), miR-27a was expressed at levels ≥2 fold lower than in CD34+ cells (Figure 1A–C). Similar trends were observed for miR-23a (Figure S3A–C) and miR-24 (Figure S3D–F). Consistent with these qRT-PCR results, miR-27a expression levels determined by microarray in a larger number of leukemia samples were significantly lower in pre-B-ALL and T-ALL cases than in CD34+ HSPCs; however, there were no significant differences in miR-27a, miR-23a, or miR-24 levels between CD34+ HSPCs and AML cases (Figure 2A–C). Expression levels of mature miR-27a, miR-23a and miR-24 were highly correlated (Figure S2B–G), and miR-27b and miR-23b expression levels were significantly lower than miR-27a and miR-23a expression in CD34+ HSPCs in all but one acute leukemia tested (Figure S4A, B), confirming a prior report that the miR-23b cluster is not expressed in hematopoietic cells [17].

### Expression of miR-27a is regulated by c-MYC

**Figure 3:**
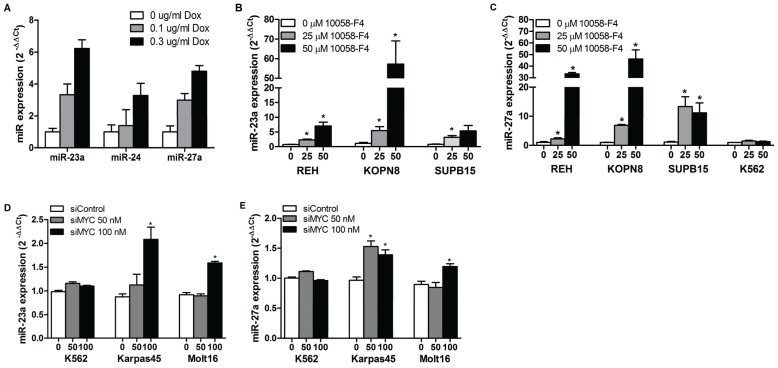
Effects of c-MYC on expression of the miR-23a cluster. (A) Knock down of c-MYC expression in P493B cells by doxycycline-treatment. Cells were treated with 0 µg/ml (white bar), 0.1 µg/ml (grey bar) or 0.3 µg/ml (black bar) doxycycline for 48 hr, harvested, and total RNA isolated. Levels of miR-23a cluster member expression were measured via qRT-PCR, using U18 as an endogenous control, and normalized to untreated (0 µg/ml Dox) cells (2^−ΔΔCt^ = 1). (B) and (C) The effects of pharmacologic inhibition of c-MYC on miR-23a (B) and miR-27a (C) in REH, KOPN8, SUPB15, and K562 cell lines were determined by treatment with 2 doses of 10058-F4 (25 µM [grey bar] and 50 µM [black bar]) or vehicle (white bar) for 48 hr. Fold-expression of mature miR-23a and miR-27a were assessed by qRT-PCR and analyzed as above. Effect of the c-MYC inhibitor on miR-23a and miR-27a expression was determined by comparison of treated cells to the mean expression (±SEM) of vehicle-treated cells Significance was determined by a Student's t-test where p<0.05 (*) indicated significance (n = 2–6 independent experiments). (D) and (E) The effect of siRNA knock-down of endogenous c-MYC on expression of miR-23a (D) and miR-27a (E) in K562, Karpas45, and Molt16 cells were assessed by transfection with 50 nM (grey bar) or 100 nM (black bar) of an siRNA-c-MYC pool or 100 nM of control siRNA (white bar). Expression levels of miR-23a and miR-27a and significance of results were assessed as in (B) and (C). n≥3 independent experiments for all samples.

MiR-23a and miR-23b expression levels were previously found to be directly negatively regulated by c-MYC [13]. To confirm that c-MYC regulation extended to the entire miR-23a cluster, we used the c-MYC-over-expressing cell line P-493B, a human B-cell lymphoma line engineered with a tet-off system [13]. MiR-23a, miR-27a and miR-24 expression levels were increased concordantly by Dox treatment of these cells (Figure 3A). Consistent with these results, treatment with a pharmacologic c-MYC inhibitor (10058-F4) increased miR-23a and miR-27a expression in REH, KOPN8, and SUPB15 human pre-B ALL cell lines by 2- to 60-fold in a dose-dependent fashion (Figure 3B, C). However, miR-27a in K562 cells was not affected by the c-MYC inhibitor (Figure 3C). Knock down of c-MYC in K562 cells did not affect miR-27a or miR-23a expression (Figure 3D, E). However, we observed a 1.3-2-fold increase in miR-27a and miR-23a expression levels in Molt16 and Karpas 45 cells upon c-MYC knock down (Figure 3D, E; Figure S5A; attempts to knock down c-MYC expression in REH, KOPN8, and SUPB15 cells were unsuccessful due to low transfection efficiencies). Finally, we observed a significant inverse correlation between c-MYC mRNA and mature miR-27a levels in human acute leukemias (Figure S5B–D); a similar inverse correlation between c-MYC and miR-23a and miR-24 was also observed (Figure S5E, F).

### MiR-27a slows growth and increases apoptosis in acute leukemia cells

**Figure 4:**
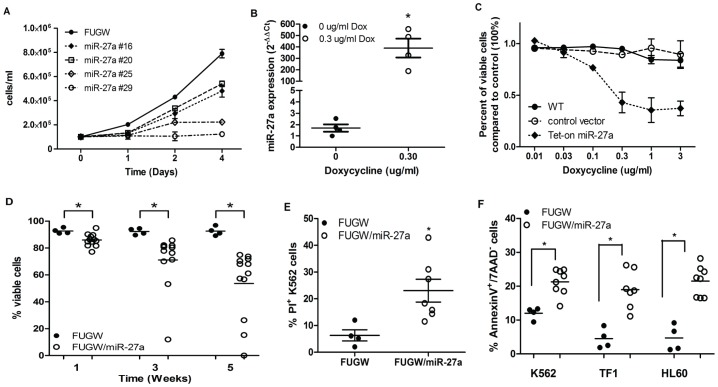
Enforced expression of miR-27a in AMLs. (A) Effects on proliferation were demonstrated by plating 10^5^ cells/ml in suspension cultures (Day 0) and counting viable cells (via trypan blue exclusion) on Days 1, 2, and 4 (n = 3 per colony). Growth was plotted as the mean viable cell number ±SD. Colony #'s were arbitrary. (B) MiR-27a expression levels in vehicle- and Dox-treated (0.30 µg/ml) TG-27a transduced K562 cells was determined by qRT-PCR and are represented as mean fold expression (2^−ΔΔCt^) ±SEM. (C) Untransduced K562 cells (WT), K562 cells transduced with a control tet-inducible plasmid, and K562 cells transduced with the TG-27a construct were treated with increasing doses of Dox (as indicated). Cell growth of control- or tet-inducible construct-transduced cells was normalized to that of untransduced K562 cells. (D) Viability of FUGW and FUGW/miR-27a transduced stable K562 clones was measured at indicated times after isolation from methylcellulose colonies. Each data point represents 1 clonal population (i.e. cells grown up from 1 colony and followed over time). Statistical differences in viability over time in FUGW and FUGW/miR-27a K562 colonies were determined by a 1-way ANOVA test (p<0.0001*). (E) FUGW and FUGW/miR-27a K562 clonal populations were stained with PI and FACS-analyzed. Each data point on the graph represents percent PI of an individual clonal population. The mean (±SEM) of FUGW clones (n = 4) was compared to FUGW/miR-27a clones (n = 7), and significant differences were assessed via student's t-test (p<0.05*). (F) K562, TF1, and HL60 AML cells were stained with AnnexinV and 7AAD and FACS-analyzed. Each data point represents the % AnnexinV^+^/7AAD^−^ population within an individual clone. Significant differences between FUGW and FUGW/miR-27a clones were compared as above.

We examined functional effects of “replacing” miR-27a expression in K562 cells by lentiviral transduction. Cells transduced with FUGW/miR-27a (GFP+) had a distinct growth disadvantage compared to control-transduced cells; in a mixed population of GFP+ and GFP− cells, the GFP+ population was reduced after 4 weeks (Figure S6A). In multiple transduced K562 cell clones, the magnitude of growth inhibition correlated with GFP and miR-27a expression levels, and FUGW/miR-27a-transduced K562 clones grew ∼2–6-fold slower in suspension cultures than controls (Figure 4A). Furthermore, no FUGW/miR-27a-transduced K562 clones could be passaged beyond ∼5–6 weeks post-transduction. To confirm these results, we engineered a tet-inducible miR-27a expression plasmid (TG-27a). K562 cells stably transduced with this plasmid expressed detectable miR-27a only upon Dox treatment (Figure 4B), and the extent of growth inhibition was dependent on Dox dose and consequent miR-27a expression level (Figure 4C).

**Figure 5:**
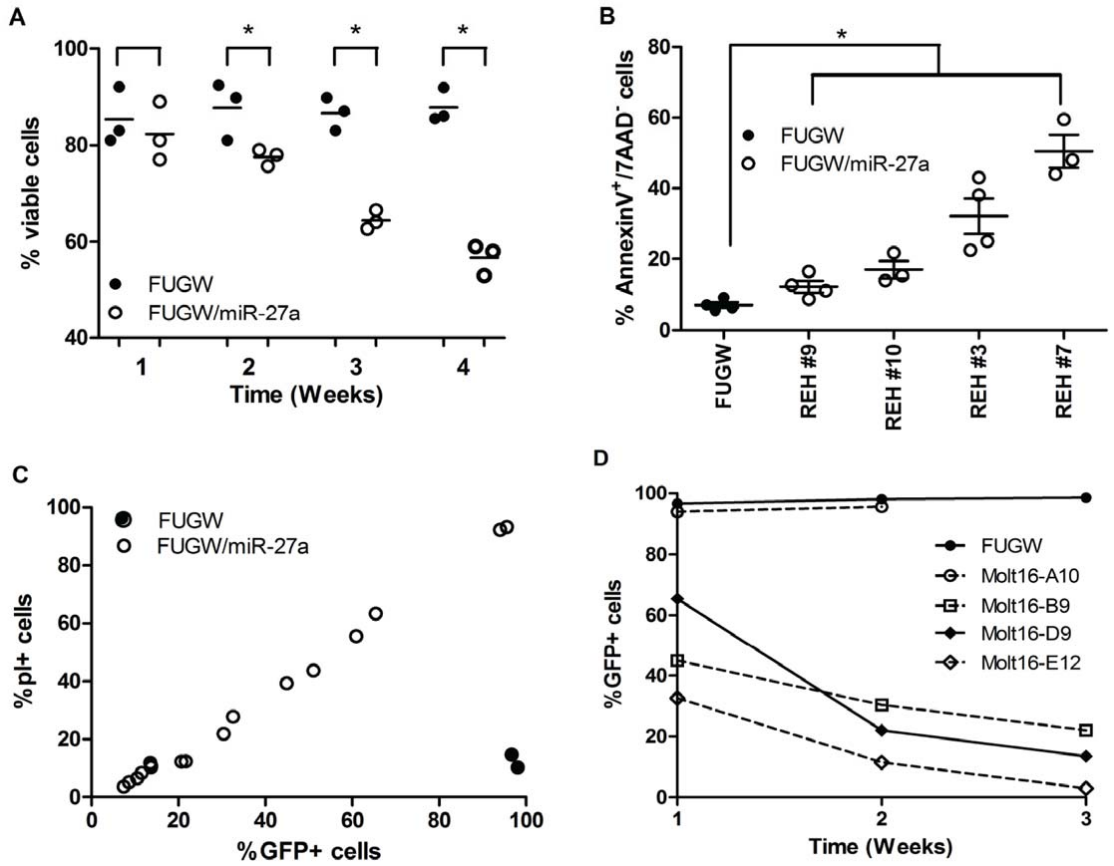
Enforced expression of miR-27a in pre-B-ALLs and T-ALLs. (A) Viability of FUGW and FUGW/miR-27a transduced REH (pre-B-ALL) clonal populations was measured at indicated times as above. Statistical differences in viability over time in FUGW and FUGW/miR-27a K562 colonies were determined by a 1-way ANOVA test (p<0.0001*). (B) REH cells were stained with AnnexinV and 7AAD and FACS-analyzed. Each data point is a replicate for the designated clonal population. Significant differences between FUGW and FUGW/miR-27a clones were compared as above. (C) FUGW and FUGW/miR-27a transduced Molt16 (T-ALL) cells were FACS-analyzed for GFP expression and PI staining. GFP expression positively correlated with PI+ cells in FUGW/miR-27a transduced cells (Pearson r = 0.9965, p<0.0001) compared to FUGW control cells which did not demonstrate this correlation. (D) GFP expression was monitored via FACS over time in FUGW and FUGW/miR-27a Molt16 GFP−/GFP+ mixed populations.

As K562 myeloid leukemia cells can generate a clonal population from a single cell, we assessed multiple stable FUGW/miR-27a and FUGW colonies. Viability of clonal populations from FUGW/miR-27a-transduced K562 colonies decreased over time as compared to cells grown from FUGW-transduced colonies which maintained viability (Figure 4D). Concurrently, there were greater numbers of PI+ cells in FUGW/miR-27a-transduced clones than in control-transduced clones (Figure 4E). There were also higher percentages of AnnexinV+/7AAD- cells in clones from FUGW/miR-27a-transduced K562 cells, as well as in FUGW/miR-27a clonal populations from other AML cell lines (TF1 and HL60), than in controls (Figure 4F). We also investigated the functional effects of miR-27a in clonal populations derived from pre-B-ALL and T-ALL cell lines. FUGW/miR-27a-transduced REH pre-B-ALL clones had lower viability over time compared to FUGW-transduced clones, similar to our observations in K562 cells (Figure 5A). FUGW/miR-27a REH clones also had higher percentages of AnnexinV+/7AAD- cells (Figure 5B, Figure S6B). For FUGW/miR-27a-transduced Molt16 T-ALL cells, only a single 100% GFP+ clonal population was isolated, and could not be passaged beyond 2 weeks. The populations of FUGW/miR-27a-transduced Molt16 cells that grew were mixed GFP+/GFP- populations; those populations with >70% GFP+ cells grew slowly (∼1 passage/week), their percentage of PI+ cells correlated with GFP (i.e. miR-27a) expression (Figure 5C, Table S2), and %GFP+ cells decreased over time, indicating a growth disadvantage (Figure 5D). We were unable to isolate individual FUGW/miR-27a clones in Karpas 45 T-ALL cells, whereas control FUGW-transduced Karpas 45 clones grew normally.

### MiR-27a negatively regulates 14-3-3θ expression

**Figure 6:**
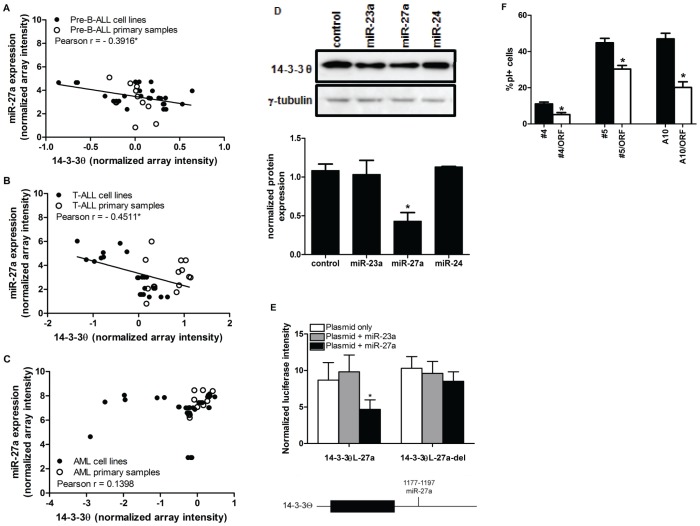
MiR-27a regulates14-3-3θ via a single binding site in the 3′UTR. (A)–(C) Expression of mature miR-27a and 14-3-3θ mRNA were measured via microarray. Correlation plots of 14-3-3θ and miR-27a expression in (A) pre-B-ALL (n = 37), (B) T-ALL (n = 26), and (C) AML (n = 39) cell lines and primary samples are shown. Significant inverse correlation was determined via Pearson r (r<**−**0.30, p<0.05*). (D) The effect of miR-27a on 14-3-3θ protein expression was measured via Western blot; the top panel is a representative experiment, and the bottom panel is a graph of normalized protein expression from 3 independent experiments (±SEM), determined by densitometry analysis (p<0.05*). (E) A schematic showing the predicted miR-27a binding site in the 3′UTR of 14-3-3θ (bottom). HEK293T cells were co-transfected with 14-3-3θL-27a or 14-3-3θL-27a-del (500 ng, white bars) alone and with 25 nM of miR-23a (grey bars) or miR-27a (black bars) (top). Normalized mean Luc expression (±SEM) is represented on the graphs. (F) The 14-3-3θ ORF rescues miR-27a-induced cell death. FUGW and FUGW/miR-27a transduced K562 clones were transfected with control plasmid (black bars) or 2 µg 14-3-3θ ORF (white bars), and stained with PI. FUGW/miR-27a transduced clones expressing the 14-3-3θ ORF had significantly less PI+ cells compared to those clones transfected with control vector. Significant differences were determined by Student's t-test (n≥3, p<0.05*).

Members of the 14-3-3 protein family are established anti-apoptotic molecules and oncogenes [18]. 5 of the 7 14-3-3 isoforms have predicted miR-23a cluster member binding sites in their 3′UTR and coding regions [19]–[22] (Table S3). The 3′UTR of 14-3-3θ contains a predicted binding site for miR-27a (Table S3, Figure S7A), and there was a moderately strong inverse correlation between 14-3-3θ mRNA and miR-27a levels in pre-B-ALLs (Figure 6A) and T-ALLs (Figure 6B), but not AMLs (Figure 6C). 14-3-3θ protein expression was lower in K562 cells transfected with artificial miR-27a mimic than with controls (Figure 6D).

We cloned the predicted miR-27a binding site of the 14-3-3θ 3′UTR downstream of Luc ORF (14-3-3θL-27a) and co-transfected the 14-3-3θL-27a plasmid, with or without miR-27a mimic, into HEK293T cells. Luc expression was significantly decreased upon co-transfection with miR-27a (Figure 6E), but not miR-23a (negative control). Deletion of 8 bases in the 14-3-3θ-27a construct complementary to the miR-27a seed region reversed the effect of miR-27a mimic on Luc expression (Figure 6E).

Finally, we cloned the 14-3-3θ ORF (without its 3′UTR) into the pcDNA3.1 expression vector. Expression of exogenous 14-3-3θ ORF was not affected by transfection of miR-27a mimic (Figure S8A, B). Over-expression of the 14-3-3θ ORF in FUGW/miR-27a-transduced K562 clones significantly decreased the percentage of PI+ cells (Figure 6F), partially rescuing the miR-27a-induced phenotype.

### MiR-27a does not regulate additional 14-3-3 isoforms

**Figure 7:**
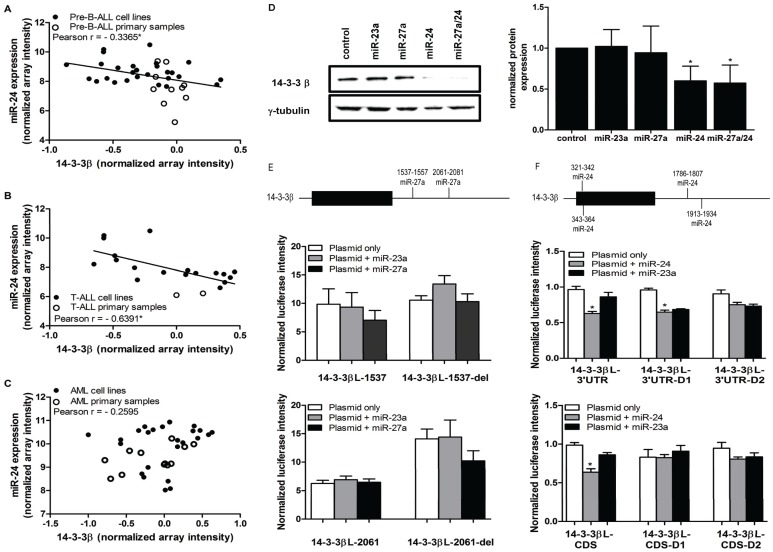
MiR-24, but not miR-27a regulates 14-3-3β via sites in the 3′UTR and CDS. (A)–(C) Expression of mature miR-24 and 14-3-3β mRNA were measured via microarray. Correlation plots of 14-3-3β and miR-24 expression in (A) pre-B-ALL (n = 37), (B) T-ALL (n = 20), and (C) AML (n = 36) cell lines and primary samples are shown. Significant inverse correlation was determined via Pearson r (r<−0.03, p<0.05*). (D) The effect of miR-23a cluster members on endogenous 14-3-3β protein expression was measured via Western blot. The left panel is a representative blot from one experiment, and the right panel is a graph of normalized protein expression from 4 independent experiments (±SEM), determined by densitometry analysis (p<0.05*). (E) A schematic showing predicted miR-27a binding sites in the 3′UTR of 14-3-3β (top). HEK293T cells were co-transfected with 14-3-3βL-1537 or 14-3-3βL-1537-del (middle panel), and 14-3-3βL-2061 or 14-3-3βL-2061-del (bottom panel) (500 ng, white bars) alone and with 25 nM of miR-23a (grey bars) or miR-27a (black bars). Normalized mean Luc expression (±SEM, n≥3) is represented on the graph. (F) A schematic shows predicted miR-24 binding sites in the CDS and 3′UTR of 14-3-3β (top). HEK293T cells were co-transfected with 14-3-3βL-3′UTR (or deletion mutants 1 and 2; middle panel) or 14-3-3βL-CDS (or deletion mutants 1 and 2; bottom panel), (500 ng, white bars) alone and with 25 nM of miR-24 (grey bars) or miR-23a (black bars). Normalized mean Luc expression (±SEM, n≥3) is represented.

14-3-3β has 2 predicted miR-27a binding sites in its 3′UTR (Table S3, Figure S7B, C). Microarray data demonstrated no correlation between miR-27a and 14-3-3β mRNA levels (data not shown). However, 14-3-3β also has 5 predicted miR-24 binding sites (Table S3, Figure S7D-G), and our microarray data indicated a significant inverse correlation between miR-24 and 14-3-3β mRNA levels in pre-B-ALL and T-ALL, but not in AML (Figure 7A–C). Transfection of K562 cells with miR-27a mimic did not affect 14-3-3β protein expression, but the miR-24 mimic significantly decreased 14-3-3β protein levels compared to control cells (Figure 7D); the combination of miR-24 and miR-27a yielded no additional decrease in endogenous 14-3-3β.

**Figure 8:**
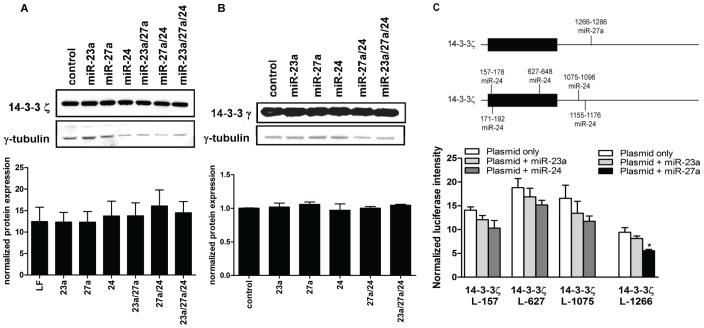
The miR-23a cluster does not regulate 14-3-3ζ, or other 14-3-3 isotypes. (A) and (B) The effect of the miR-23a cluster on (A) 14-3-3ζ and (B) 14-3-3γ protein expression was measured via Western blot. The top panels are representative blots from one experiment, and the bottom panels are graphs of normalized protein expression from 3 independent experiments (±SEM), determined by densitometry analysis (p<0.05*). (C) The schematics show predicted miR-27a and miR-24 binding sites in the CDS and 3′UTR of 14-3-3ζ (top panel). HEK293T cells were co-transfected with 14-3-3ζL-157, 14-3-3ζL-627, or 14-3-3ζL-1075 (500 ng, white bars) alone and with 25 nM of miR-23a (light grey bars) or miR-24 (dark grey bars); HEK293 cells were also co-transfected with 14-3-3ζL-1266 (500 ng, white bars) alone and with 25 nM of miR-23a (light grey bars) or miR-27a (black bars) (bottom panel). Normalized mean Luc expression (±SEM, n≥3) is represented on the graphs.

To determine the miR binding sites necessary for regulation of 14-3-3β protein expression, we cloned the 2 miR-27a sites (individually, 14-3-3βL-1537 and 14-3-3βL-2061) and 4 of the 5 miR-24 sites (14-3-3βL-3′UTR and 14-3-3βL-CDS) into our Luc reporter vector (Table S3, Figure S7B–G). Co-transfection of miR-27a plus 14-3-3βL-1537 or 14-3-3βL-2061 resulted in no significant Luc reduction compared to miR-23a or plasmid alone (Figure 7E). In contrast, co-transfection of miR-24 plus 14-3-3βL-3′UTR reduced Luc expression compared to miR-23a or plasmid alone. Deletion of the seed complement in site 1 had no effect on Luc expression; however, Luc inhibition was partially reversed in the presence of miR-24 upon deletion of the seed complement in both sites (Figure 7F). Co-transfection of miR-24 plus 14-3-3βL-CDS resulted in significantly reduced Luc expression compared to miR-23a or plasmid only (Figure 7F). Deletion of the seed bases in site 1 completely reversed inhibition of Luc activity; however, deletion of the seed in both sites resulted in some (p>0.05) retained Luc reduction in the presence of both miR-24 and miR-23a; this inhibition may be non-specific. The last miR-24 site was the least thermodynamically strong of the predicted binding sites, and as 3 of the 4 sites previously cloned demonstrated miR-24 binding, we did not clone this last predicted miR-24 site.14-3-3ζ and 14-3-3γ each have multiple predicted miR-23a cluster binding sites (Table S3, Figure S7H–M). However, there was no significant inverse correlation between 14-3-3ζ mRNA expression and either miR-27a or miR-24 (data not shown); the same was true for 14-3-3γ and miR-27a, with the exception of a slight inverse correlation in T-ALL samples, and for 14-3-3γ and miR-24 (data not shown). K562 cells transfected with the miR-23a cluster mimics, alone or in combination, did not have decreased endogenous 14-3-3ζ or 14-3-3γ protein expression compared to control (Figure 8A, B). We cloned predicted miR-27a and miR-24 sites of 14-3-3ζ into our Luc reporter plasmid, and detected little or no reduction of Luc activity upon co-transfection of these plasmids with miR-27a or miR-24 (Figure 8C). In addition, we examined the effect of the miR-23a cluster members on 14-3-3ε protein expression (no predicted miR-23a cluster binding sites) in K562 cells; as expected, none of these miRs had a significant effect on 14-3-3ε protein expression by Western blotting (data not shown).

## Discussion

We found that miR-27a, like its coordinately expressed miRs, was expressed at high levels in normal human CD34+ HSPCs. MiR-27a expression strongly correlated with expression of miR-23a and miR-24 in CD34+ HSPCs, consistent with coordinated regulation of other miR clusters. The miR-23a cluster has been reported as either up- or down-regulated in disease, suggesting tissue specific regulation [13], and cooperative functioning of this cluster in physiology and pathophysiology [13]. Although there is a paralogous miR-23b cluster, it is expressed at very low levels in hematopoietic cells [17], as confirmed by our studies. Specifically, miR-27a was expressed ≥4-fold higher in CD34+ HSPCs.

In contrast to high expression of the miR-23a cluster in CD34+ HSPCs, thought to include the cells of origin of acute leukemias [23]–[25], expression of miR-27a was significantly decreased in 64–66% of acute leukemia cell lines and primary samples examined by qRT-PCR or microarray. 97% of samples with low miR-27a had significantly down-regulated miR-23a and miR-24. This down-regulation was present in >80% of pre-B-ALL and T-ALL cell lines and primary samples tested, but in fewer AML samples, consistent with previous reports that the miR-23a cluster is more highly expressed in AML than ALL [26]–[28]. A recent study demonstrating that down-regulation of miR-27a expression in childhood pre-B-ALL correlates with disease relapse, and that patients achieving complete remission had high levels of miR-27a expression complement our findings [29]. This study also reported that patients expressing high levels of miR-27a had a higher relapse-free survival rate of than patients with low levels [29]. In addition, a recent analysis of >4,000 normal and neoplastic human samples reported that the miR-23a and miR-23b clusters are among those genetically deleted in human cancers, including lung cancer, prostate cancer, and CLL [30].

We observed an inverse correlation between levels of c-MYC mRNA and miR-27a, miR-23a, and miR-24 expression in normal CD34+ HSPCs and acute leukemias, suggesting that c-MYC contributes to the regulation of the miR-23a cluster in the hematopoietic system. Gao et al [14] showed that c-MYC down-regulated expression of miR-23a and miR-23b and bound to a cognate site upstream of the miR-23b locus in a B lymphoma cell line, others have provided evidence for a feedback loop involving direct negative regulation of c-MYC by miR-24 [13], [31], and there is growing evidence for regulation of miRs by c-MYC as well as regulation of c-MYC by miRs in cancers including leukemia [32], [33]. Using a conditional c-MYC expressing lymphoma cell line, we showed that miR-27a and miR-24 were coordinately regulated, along with miR-23a, by c-MYC. However, although our profiling data demonstrates coordinate regulation of all 3 cluster members, due to the fact that miR-24-2 and miR-24-1 have the same sequence, we cannot definitively say that miR-24-2 is the only contributor to this expression change in P-493B cells. In addition, a small molecule inhibitor of c-MYC, 10058-F4, which disrupts the interaction between c-MYC and its heterodimeric partner, MAX, thus preventing the complex from binding to a canonical E-Box DNA binding site, up-regulated miR-23a and miR-27a expression in 3 of 4 acute leukemia cell lines tested. One such site is present ∼1000 bp upstream of the miR-23a cluster [11], [34]–[36]; (Figure S1). Thus, evidence suggests that the miR-23a cluster is transcriptionally regulated by c-MYC and is not genetically deleted in the majority of acute leukemias. The dose of 10058-F4 used in our studies (25–50 uM) was chosen based on previously published experiments; functional studies in leukemia cell lines used 100 µM to inhibit c-MYC, and 60 µM of 10058-F4 was used to demonstrate that c-MYC inhibition increased levels of let-7a, let-7b, and miR-98 expression, mimicking the effects of siRNA knock down of c-MYC [37]–[39]. We verified the results using 10058-F4 by siRNA-mediated c-MYC knock-down in 3 cell lines, which resulted in a 2-fold increase in both miR-23a and miR-27a in 2 of 3 lines. Failure of either siRNA knock-down of c-MYC or 10058-F4 to affect miR-23a and miR-27a expression in K562 cells suggests additional mechanisms of miR-23a cluster regulation. Defined sites of DNA methylation are present in a 500 bp region upstream of the miR-23a cluster in K562 cells, suggesting possible epigenetic regulation in these cells [40]. Additionally, the miR-23a promoter contains a response element for the promyelocytic leukemia gene-retinoic acid receptor alpha (PML-RARA) fusion protein (Figure S1), and chromosomal immunoprecipitation studies have identified involvement of PML, RARA and retinoid-X receptor alpha in regulation of this cluster [41]. Interestingly, qRT-PCR and microarray expression data indicated that miR-23a cluster member expression was not decreased in HL60, a myeloid cell line with some morphologic features of promyelocytic leukemia that lacks PML-RARA [41].

Based on the differential expression of miR-27a in leukemias versus CD34+ HSPCs, we hypothesized that miR-27a might have tumor suppressor functionality. To test this hypothesis, we “replaced” miR-27a in human acute leukemia cell lines. Constitutive expression of miR-27a at levels comparable to endogenous CD34+ HSPC levels resulted in immediate growth inhibition and cell death in AML, pre-B-ALL, and T-ALL cell lines. None of these cell lines survived for more than ∼15 population doublings upon miR-27a replacement; in neither of the 2 T-ALL cells lines tested were we able to successfully isolate clonal populations of miR-27a-expressing cells.

This growth inhibition was partially due to increased apoptosis and cell death, observed in all 5 human leukemia cell lines tested; ”replacement” of miR-27a induced a 10–20% increase in apoptotic K562, HL-60, TF1, and REH cells, and up to 80% dead (PI+) Molt16 cells. Induction of apoptosis in HEK293T cells upon expression of the 3 miR-23a cluster members together has been reported [42], and miR-24 over-expression, alone and in combination with cisplatin or etoposide, induced apoptosis in MCF7 breast cancer cells [43], [44], but apoptosis induced specifically by miR-27a or miR-23a has not been shown. However, miR-27a expression is down-regulated in drug-resistant K562 and HL60 cells lines, and over-expression of miR-27a increased the sensitivity of these resistant cell lines to doxorubicin [45]. Induction of apoptosis is a common result of replacement of tumor suppressor miRs, as found for miR-15a∼miR-16-1 in CLL [5], miR-34 in CLL [46], neuroblastoma [47], and prostate cancer [48] cell lines, and miR-29b in AML [49].

In the miR-27a predicted target lists [19]–[22], we noted several 14-3-3 isoforms. The 14-3-3 family includes 7 isoforms with 70–80% amino acid homology which bind phosphorylated serine and threonine residues to regulate multiple cellular processes, including cell cycle, apoptosis, and signal transduction [18]. 14-3-3 proteins are dysregulated in cancers [18] and can act as either tumor suppressors (14-3-3σ) or oncogenes (14-3-3β, ζ, γ, or θ) by binding and sequestering phosphorylated pro-apoptotic proteins such as BAX, BAD, ASK1, Foxo1 or Foxo3a [18].

We observed a moderate inverse correlation between mature miR-27a expression and 14-3-3θ mRNA levels across the acute leukemias. Additionally, via reporter assays and Western blots, we demonstrated direct down-regulation of 14-3-3θ by miR-27a, dependent on a 3′UTR binding site. Lack of higher correlation between miR-27a and 14-3-3θ mRNA expression may reflect a predominantly translational inhibitory mechanism [3], [4]. To demonstrate a direct relationship between miR-27a-induced cell death/apoptosis and down-regulation of 14-3-3θ, we over-expressed the 14-3-3θ ORF (to render it “insensitive” to miR-27a). The ORF was able to partially rescue miR-27a-induced cell death increase, indicating that 14-3-3θ contributes to miR-27a function, and that there are likely additional miR-27a target genes that play a role in miR-27a-induced cell death. In contrast, we were surprised to find that miR-27a does not regulate 14-3-3β, by Western blotting or Luc assays, despite 2 predicted binding sites in its 3′UTR. However, we found miR-24 down-regulated 14-3-3β expression via predicted sites within the CDS and the 3′UTR. When 14-3-3β is decreased, pro-apoptotic proteins, such as BAD, are released and translocate to the mitochondria to induce apoptotic signaling [18], [50]. Apoptosis due to miR-24 mediated down-regulation of BCL2 has been reported and reduction of 14-3-3β further contributes to apoptosis. Regulation of 14-3-3β by a CDS miR site emphasizes the need to take the CDS into account when assessing miR targets. Similarly, Nanog, Oct4, and Sox2 have confirmed miR target sites in their CDSs [51]. A recent report [31] demonstrated that multiple miR-24-regulated genes have “seedless”, but otherwise highly complementary binding sites. We were surprised to observe that neither miR-27a nor miR-24 regulated 14-3-3ζ or 14-3-3γ, despite multiple predicted binding sites. This observation highlights the high false positive rate of miR target prediction algorithms. As expected, expression of 14-3-3ε protein, our negative control containing no predicted miR-27a sites, was not affected by the miR-23a cluster.

The observation that miR-27a and miR-24 each potentially target a different 14-3-3 isoform suggests that these miRs may cooperate to down-regulate pro-apoptotic members of the 14-3-3 gene family. Multiple reports indicate that efficient knockdown of a single 14-3-3 family member, by an isoform-specific siRNA, is sufficient to induce functional changes, including increasing apoptosis; knock-down of 14-3-3θ induced release and translocation of BAD to the mitochondria [52], and siRNA-directed knock-down of 14-3-3γ up-regulated BAD and BAX and induced apoptosis in BAF3 and K562 cells [53]. Conversely, over-expression of a single isoform (i.e. 14-3-3θ) was sufficient to inhibit apoptosis [54]. Thus, a significant pro-apoptotic effect might be accomplished even by inefficient down-regulation of individual 14-3-3 isoforms by the actions of multiple miRs acting in concert. Further studies would be needed to definitively confirm a synergistic or additive effect between miR-27a and miR-24 in inducing apoptosis in acute leukemias via regulation of 14-3-3 family members. Croce and colleagues have demonstrated such coordinated action in the miR-15a∼miR-16-1 cluster in down-regulation of BCL-2 in CLL [55].

In summary, we confirmed that miR-27a expression is down-regulated in multiple acute leukemia cell lines and primary patient samples. Enforced expression (“replacement”) of miR-27a resulted in decreased cell growth and increased cell death due, at least in part, to an increase in cellular apoptosis. MiR-27a-induced apoptosis occurred via specific down-regulation of 14-3-3θ, confirmed by the fact that functional effects were “rescued” by miR-27a-resistant 14-3-3θ ORF. Further, binding and down-regulation of 14-3-3β by miR-24, which was also significantly down-regulated in acute leukemias, indicates that a second miR-23a cluster member may have a similar functional effect, although we did not conduct ORF rescue experiments with miR-24 and 14-3-3β. Thus, miR-27a plays a tumor suppressor-like role in acute leukemias by regulating apoptosis, and miR-27a and 14-3-3θ are potential target molecules for development of leukemia therapeutics.

## Supporting Information

Figure S1 **Schematics and mature sequences of the miR-23a and miR-23b cluster loci:** The miR-23a cluster is located on the negative strand of human chromosome 19 (19p13.2), and has a defined transcription start site at −600 to +32 bp upstream of the open reading frame. Within this region, there are also canonical c-MYC and PML-RARA binding sites defined (11–14, 41). The cluster is intergenic and lies between the genes ZSWIM4 and NANOS3 both of which are on the positive strand; miR-181c and miR-181d are located between the miR-23a cluster and NANOS3, also on the positive strand. Cluster organization is syntenic to both mouse and rat. The miR-23b cluster is located on human chromosome 9 (9q22.32) on the positive strand. The cluster is intragenic, located within the C9orf3 gene and is transcribed from its own promoter approximately 31 Kb upstream. The sequences of miR-23a and miR-23b differ by only one base (position 19, U vs. A) as do miR-27a and miR-27b (position 19, C vs. U); miR-24-2 and miR-24-1 have identical mature sequences.
(TIF)

Figure S2 **MiR-23a cluster expression in normal CD34+ HSPCs.** (A) Mature miR-23a (n = 33), miR-27a (n = 36), miR-24 (n = 24) and U18 control (n = 30) levels were determined using specific TaqMan qRT-PCR and presented as the raw Ct value. (B)–(G) Expression of miR-27a correlated with miR-23a and miR-24 expression in normal CD34+ HSPCs and acute leukemia cell lines and primary samples, as determined by qRT-PCR (B)–(D) and microarray analysis (E)–(G). Mature miR-27a, miR-23a and miR-24 expression levels were measured via qRT-PCR and are expressed as raw Ct values on the graphs. Correlation of miR-27a to miR-23a (B) (n = 108), miR-27a to miR-24 (C) (n = 99), and miR-23a to miR-24 (D) (n = 99) were both positive and significant (Pearson r>0.900, p<0.05*). Expression levels of mature miR-27a, miR-23a, and miR-24 were measured by microarray analysis and expressed as their normalized array intensities on the graphs. Correlation of miR-27a to miR-23a (E) (n = 133), miR-27a to miR-24 (F) (n = 133), and miR-23a to miR-24 (G) (n = 133) were also both positive and significant (Pearson r>0.85, p<0.05*).
(TIF)

Figure S3 **Levels of mature miR-23a and miR-24 expression in acute leukemias.** MiR-23a and miR-24 expression was measured in AML (A, D), pre-B-ALL (B, E), and T-ALL (C, F) cell lines and primary samples. MiR-23a and miR-24 levels in cell lines (black bars) and primary samples (white bars) were determined, from total RNA enriched for small RNAs, by specific TaqMan qRT-PCR analysis and expression presented as mean 2^−ΔΔCt^ (±SEM) normalized to the level of miR-23a in CD34+ HSPCs (2^−ΔΔCt^ = 0.9744±0.034) or miR-24 in CD34+ HSPCs (2^−ΔΔCt^ = 0.9959±0.029), as in Figure 1. Significance was determined via student's t-test and indicated at p<0.05*; n≥3 independent experiments.
(TIF)

Figure S4 **Levels of mature miR-27b and miR-23b expression in acute leukemias.** MiR-27b (A) and miR-23b (B) were significantly lower than mature miR-27a and miR-23a, respectively, in normal human CD34+ HSPCs and acute leukemias. Total RNA, enriched for small RNAs, was analyzed by qRT-PCR, and fold expression levels are presented (2^−ΔΔCt^) normalized to the level of miR-27a (2^−ΔΔCt^ = 0.9756±0.022) or miR-23a (2^−ΔΔCt^ = 0.9744±0.034) expression in CD34+ HSPCs. Significant differences between expression levels of “a” miRs (white bars) compared to “b” miRs (black bars) was assessed by Student's t-test; significance was indicated where p<0.05*; for all samples, n≥3 independent experiments.
(TIF)

Figure S5 **The effects of c-MYC on miR-23a cluster expression**. (A) K562, Molt16, and Karpas45 cells were transfected with an siRNA-c-MYC pool or control siRNA as described, and c-MYC protein expression was assessed via Western blot. (B)–(D) Expression of c-MYC mRNA and mature miR-27a was measured via microarray, and correlation plots of c-MYC and miR-27a expression in (B) pre-B-ALL (n = 37), (C) T-ALL (n = 18), and (D) AML (n = 33) cell lines and primary samples are shown. Significant inverse correlation was determined via Pearson r (r<−0.3, p<0.05*). (E, F) Expression of c-MYC mRNA and miR-23a (E) or miR-24 (F) was measured as in (B–D) and cumulative correlation plots including AML, pre-B-ALL, and T-ALL samples are shown. As above, significant inverse correlation was determined via Pearson r.
(TIF)

Figure S6 **MiR-27a induces a growth disadvantage and increase in cell death.** (A) A mixed population of FUGW/miR-27a transduced GFP+ (i.e. miR-27a expressing) K562 cells and untransduced GFP- K562 cells were analyzed by FACS over time. Cell number is represented on the y-axis, and GFP intensity is represented on the x-axis. (B) These representative FACS plots show AnnexinV on the X-axis and 7AAD on the y-axis. Cells undergoing active apoptosis are present in the lower right quadrant of these plots, corresponding to those cells that stain AnnexinV+/7AAD-.
(TIF)

Figure S7 **Sequence alignments of mature miRs with their predicted binding sites in 14-3-3 proteins.** (A)–(M) Sequence alignments correspond to the predicted miR binding sites cloned into the pcDNA3.1-Luc reporter plasmid. Bases underlined and in italics represent target gene bases complementary to the miR seed region. These bases were deleted to abolish miR binding.
(TIF)

Figure S8 **MiR-27a does not regulate 14-3-3θ without its 3′UTR.** (A) The effect of miR-27a on 14-3-3θ ORF expression was measured via Western blot; Lane 1, control (endogenous 14-3-3θ); Lane 2, 14-3-3θ ORF; Lane 3, 14-3-3θ ORF +50 nM miR-23a; Lane 4, 14-3-3θ ORF +50 nM miR-27a. (B) Densitometry analysis of 2 replicates of the above Western blot normalized to γ-tubulin.
(TIF)

Table S1 **Primer sets used to clone predicted miR binding sites into the Luc reporter plasmid and to delete the seed region complement**.
(DOCX)

Table S2 **Correlation of GFP^+^ expression and pI^+^-staining cells in Molt16 FUGW/miR-27a-transduced cells.**
(DOCX)

Table S3 **Predicted binding sites for miR-23a cluster members in 14-3-3 isoforms.**
(DOCX)
