# Supplementary figures and images for: MiR-27a Functions as a Tumor Suppressor in Acute Leukemia by Regulating 14-3-3θ
Source: PLoS One. 2012 Dec 7;7(12):e50895. doi: 10.1371/journal.pone.0050895 (PMC3517579; doi:10.1371/journal.pone.0050895)

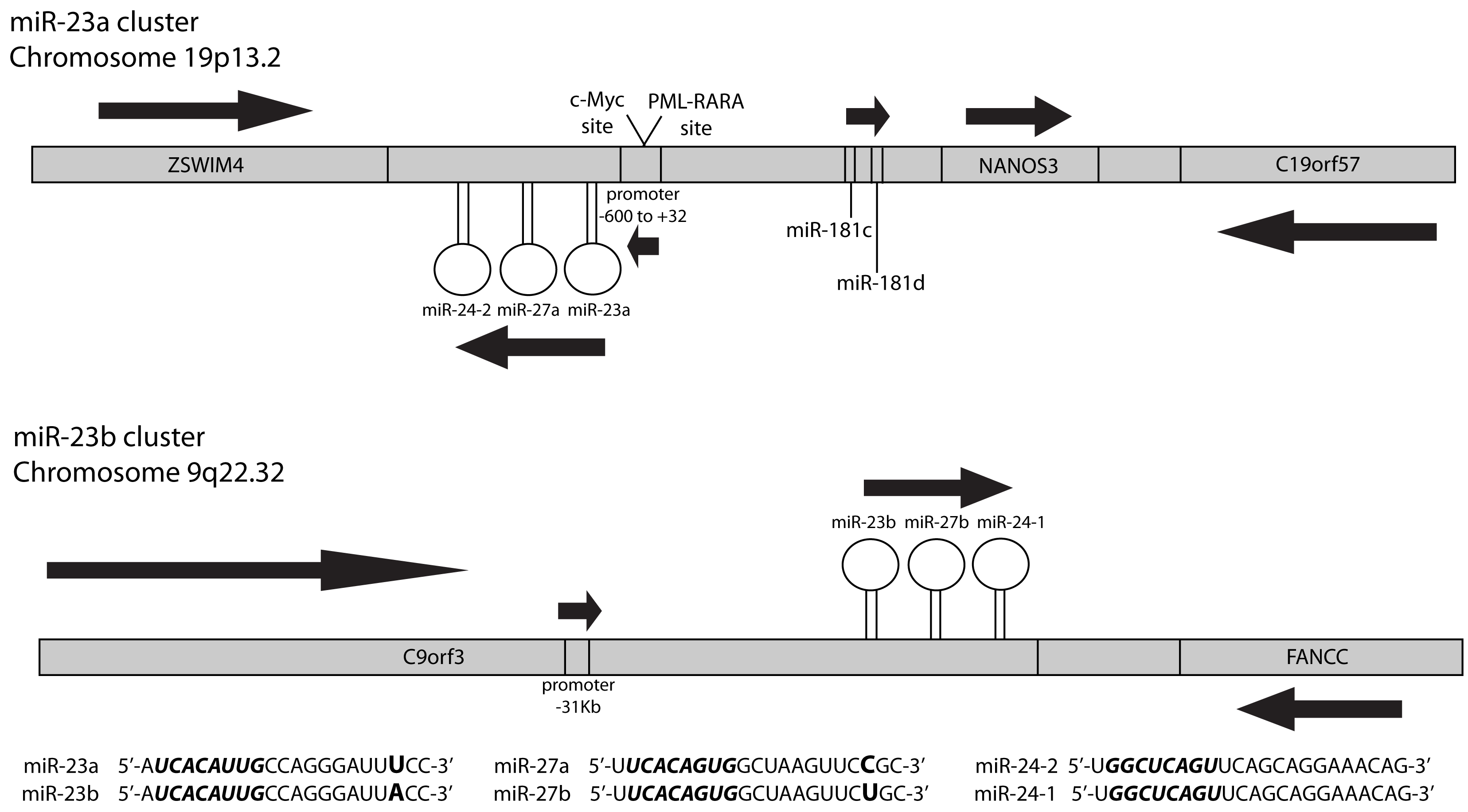

Supplement: Figure S1 — Schematics and mature sequences of the miR-23a and miR-23b cluster loci: The miR-23a cluster is located on the negative strand of human chromosome 19 (19p13.2), and has a defined transcription start site at −600 to +32 bp upstream of the open reading frame. Within this region, there are also canonical c-MYC and PML-RARA binding sites defined (11–14, 41). The cluster is intergenic and lies between the genes ZSWIM4 and NANOS3 both of which are on the positive strand; miR-181c and miR-181d are located between the miR-23a cluster and NANOS3, also on the positive strand. Cluster organization is syntenic to both mouse and rat. The miR-23b cluster is located on human chromosome 9 (9q22.32) on the positive strand. The cluster is intragenic, located within the C9orf3 gene and is transcribed from its own promoter approximately 31 Kb upstream. The sequences of miR-23a and miR-23b differ by only one base (position 19, U vs. A) as do miR-27a and miR-27b (position 19, C vs. U); miR-24-2 and miR-24-1 have identical mature sequences. (TIF) [file pone.0050895.s001.tif]

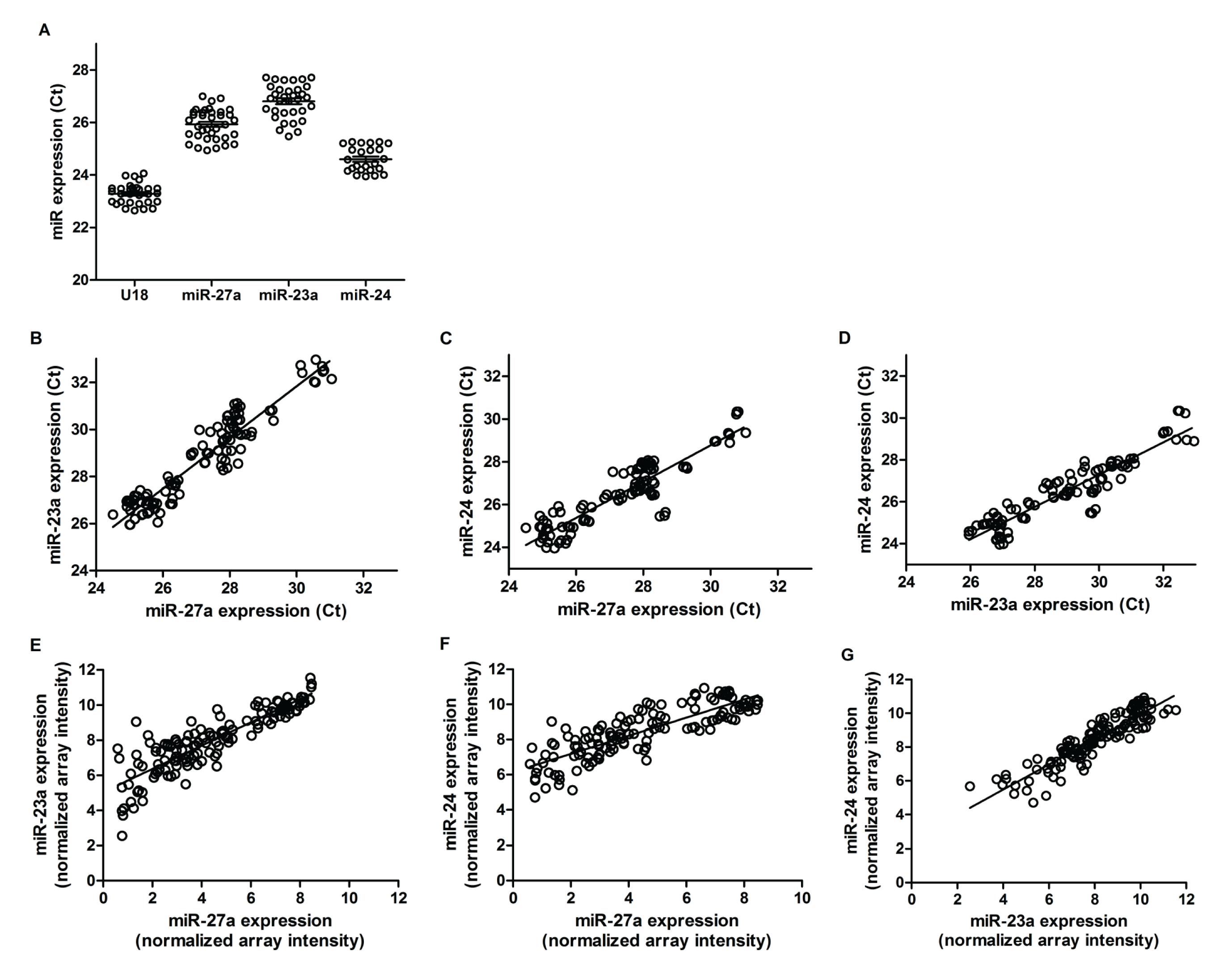

Supplement: Figure S2 — MiR-23a cluster expression in normal CD34+ HSPCs. (A) Mature miR-23a (n = 33), miR-27a (n = 36), miR-24 (n = 24) and U18 control (n = 30) levels were determined using specific TaqMan qRT-PCR and presented as the raw Ct value. (B)–(G) Expression of miR-27a correlated with miR-23a and miR-24 expression in normal CD34+ HSPCs and acute leukemia cell lines and primary samples, as determined by qRT-PCR (B)–(D) and microarray analysis (E)–(G). Mature miR-27a, miR-23a and miR-24 expression levels were measured via qRT-PCR and are expressed as raw Ct values on the graphs. Correlation of miR-27a to miR-23a (B) (n = 108), miR-27a to miR-24 (C) (n = 99), and miR-23a to miR-24 (D) (n = 99) were both positive and significant (Pearson r>0.900, p<0.05*). Expression levels of mature miR-27a, miR-23a, and miR-24 were measured by microarray analysis and expressed as their normalized array intensities on the graphs. Correlation of miR-27a to miR-23a (E) (n = 133), miR-27a to miR-24 (F) (n = 133), and miR-23a to miR-24 (G) (n = 133) were also both positive and significant (Pearson r>0.85, p<0.05*). (TIF) [file pone.0050895.s002.tif]

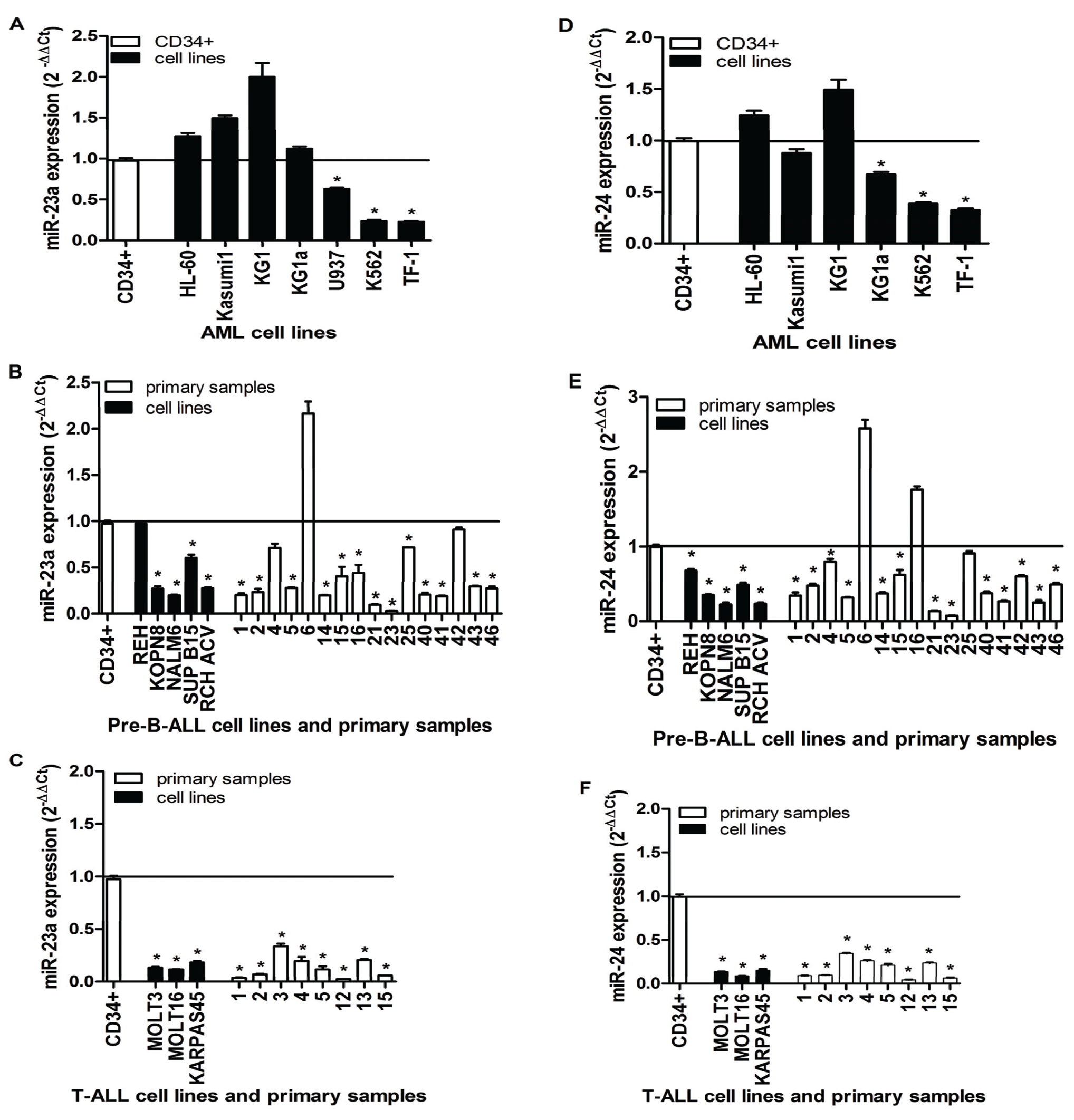

Supplement: Figure S3 — Levels of mature miR-23a and miR-24 expression in acute leukemias. MiR-23a and miR-24 expression was measured in AML (A, D), pre-B-ALL (B, E), and T-ALL (C, F) cell lines and primary samples. MiR-23a and miR-24 levels in cell lines (black bars) and primary samples (white bars) were determined, from total RNA enriched for small RNAs, by specific TaqMan qRT-PCR analysis and expression presented as mean 2−ΔΔCt (±SEM) normalized to the level of miR-23a in CD34+ HSPCs (2−ΔΔCt = 0.9744±0.034) or miR-24 in CD34+ HSPCs (2−ΔΔCt = 0.9959±0.029), as in Figure 1. Significance was determined via student's t-test and indicated at p<0.05*; n≥3 independent experiments. (TIF) [file pone.0050895.s003.tif]

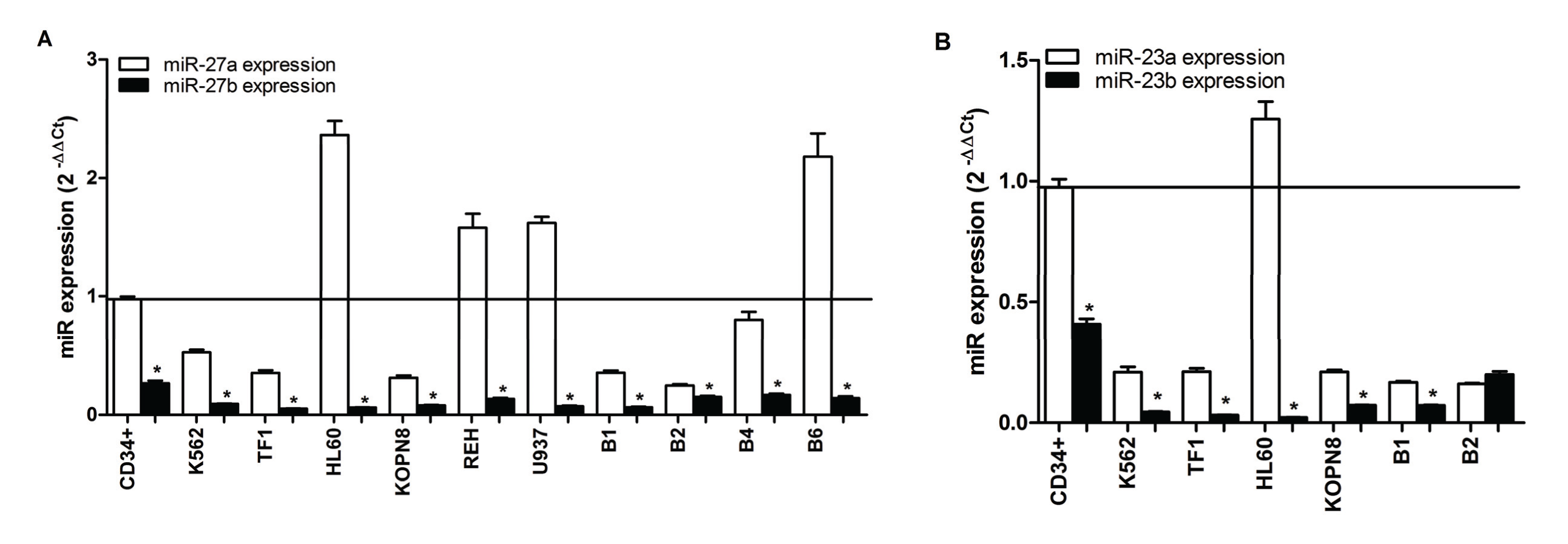

Supplement: Figure S4 — Levels of mature miR-27b and miR-23b expression in acute leukemias. MiR-27b (A) and miR-23b (B) were significantly lower than mature miR-27a and miR-23a, respectively, in normal human CD34+ HSPCs and acute leukemias. Total RNA, enriched for small RNAs, was analyzed by qRT-PCR, and fold expression levels are presented (2−ΔΔCt) normalized to the level of miR-27a (2−ΔΔCt = 0.9756±0.022) or miR-23a (2−ΔΔCt = 0.9744±0.034) expression in CD34+ HSPCs. Significant differences between expression levels of “a” miRs (white bars) compared to “b” miRs (black bars) was assessed by Student's t-test; significance was indicated where p<0.05*; for all samples, n≥3 independent experiments. (TIF) [file pone.0050895.s004.tif]

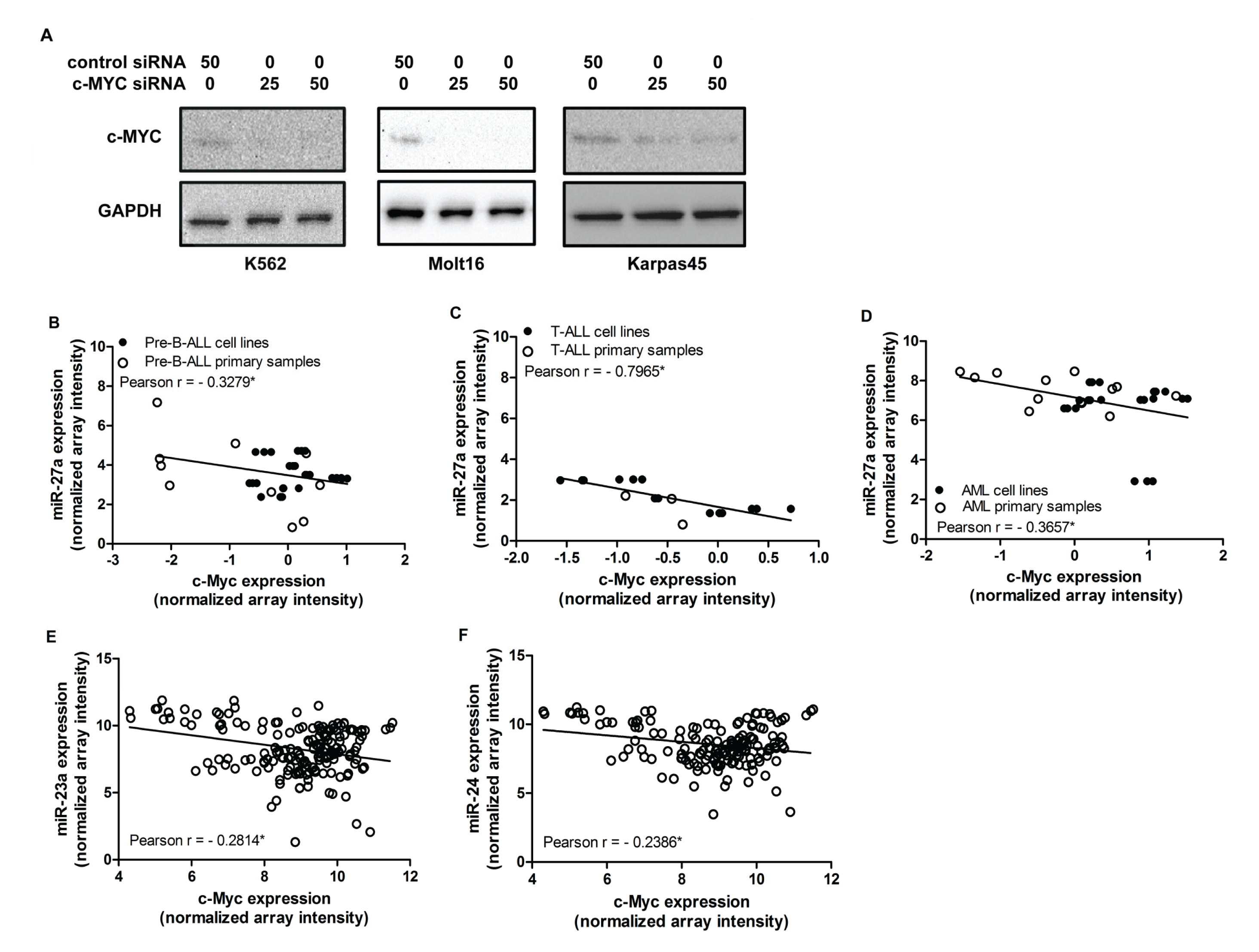

Supplement: Figure S5 — The effects of c-MYC on miR-23a cluster expression. (A) K562, Molt16, and Karpas45 cells were transfected with an siRNA-c-MYC pool or control siRNA as described, and c-MYC protein expression was assessed via Western blot. (B)–(D) Expression of c-MYC mRNA and mature miR-27a was measured via microarray, and correlation plots of c-MYC and miR-27a expression in (B) pre-B-ALL (n = 37), (C) T-ALL (n = 18), and (D) AML (n = 33) cell lines and primary samples are shown. Significant inverse correlation was determined via Pearson r (r<−0.3, p<0.05*). (E, F) Expression of c-MYC mRNA and miR-23a (E) or miR-24 (F) was measured as in (B–D) and cumulative correlation plots including AML, pre-B-ALL, and T-ALL samples are shown. As above, significant inverse correlation was determined via Pearson r. (TIF) [file pone.0050895.s005.tif]

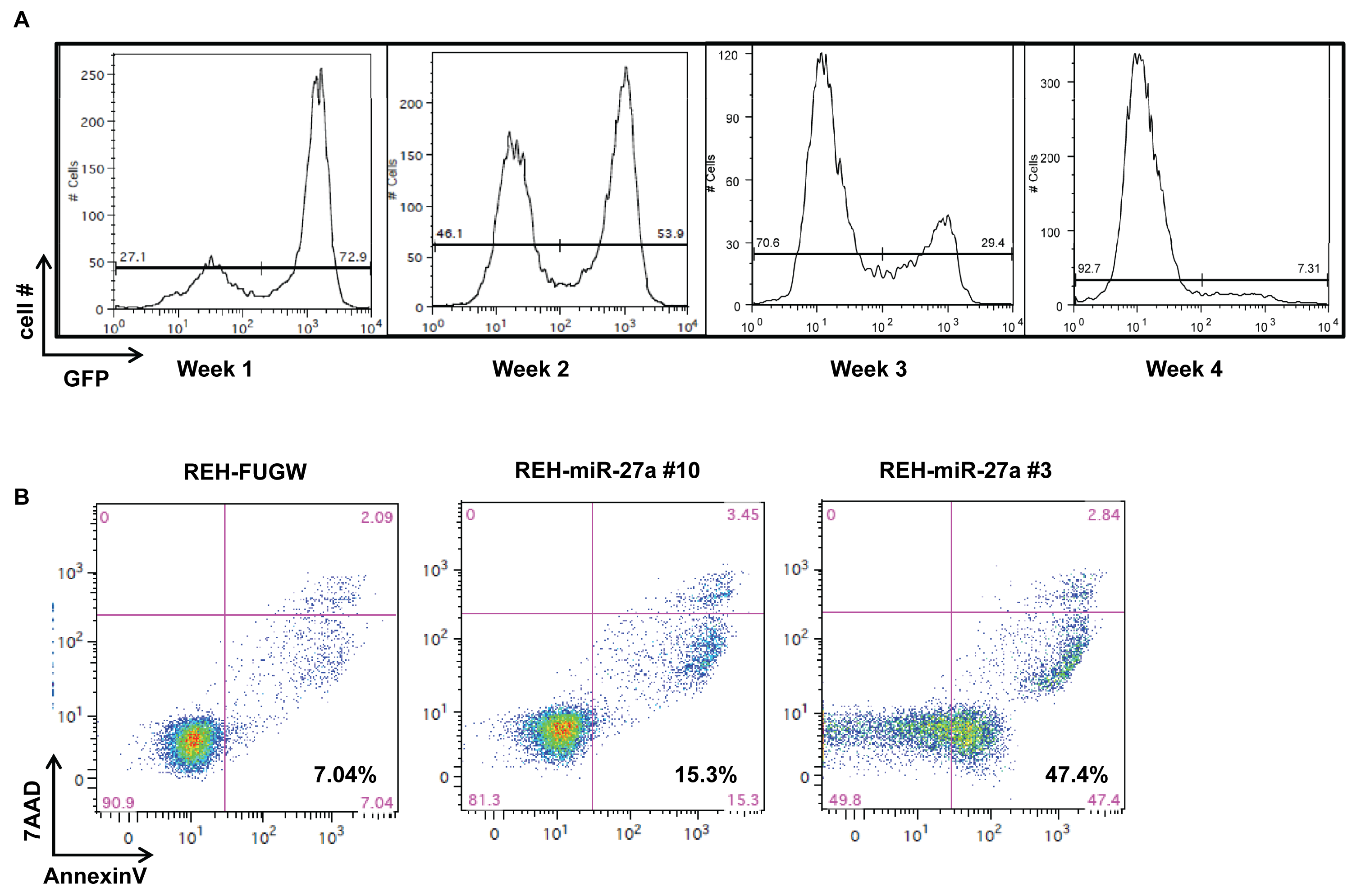

Supplement: Figure S6 — MiR-27a induces a growth disadvantage and increase in cell death. (A) A mixed population of FUGW/miR-27a transduced GFP+ (i.e. miR-27a expressing) K562 cells and untransduced GFP- K562 cells were analyzed by FACS over time. Cell number is represented on the y-axis, and GFP intensity is represented on the x-axis. (B) These representative FACS plots show AnnexinV on the X-axis and 7AAD on the y-axis. Cells undergoing active apoptosis are present in the lower right quadrant of these plots, corresponding to those cells that stain AnnexinV+/7AAD-. (TIF) [file pone.0050895.s006.tif]

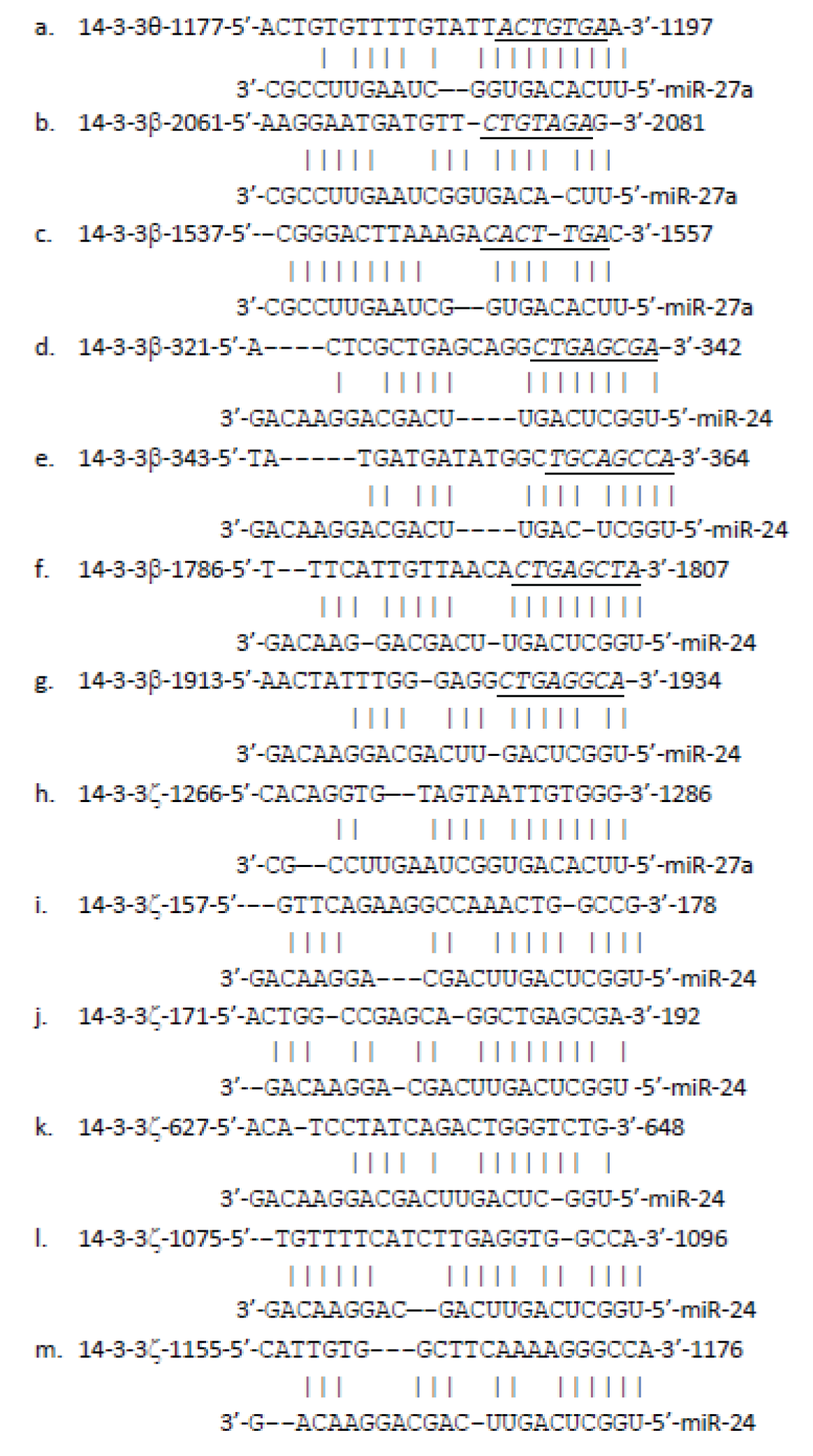

Supplement: Figure S7 — Sequence alignments of mature miRs with their predicted binding sites in 14-3-3 proteins. (A)–(M) Sequence alignments correspond to the predicted miR binding sites cloned into the pcDNA3.1-Luc reporter plasmid. Bases underlined and in italics represent target gene bases complementary to the miR seed region. These bases were deleted to abolish miR binding. (TIF) [file pone.0050895.s007.tif]

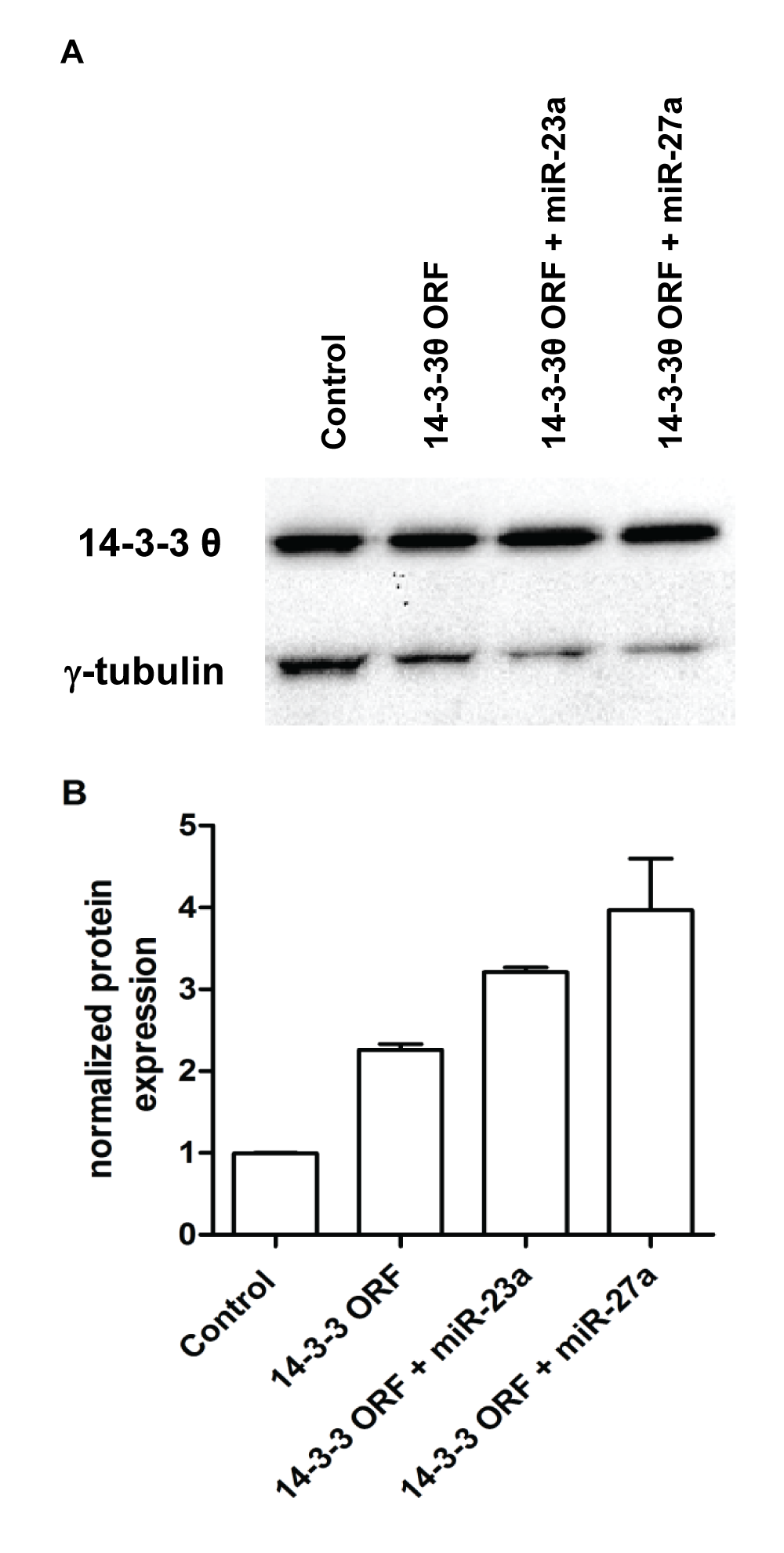

Supplement: Figure S8 — MiR-27a does not regulate 14-3-3θ without its 3′UTR. (A) The effect of miR-27a on 14-3-3θ ORF expression was measured via Western blot; Lane 1, control (endogenous 14-3-3θ); Lane 2, 14-3-3θ ORF; Lane 3, 14-3-3θ ORF +50 nM miR-23a; Lane 4, 14-3-3θ ORF +50 nM miR-27a. (B) Densitometry analysis of 2 replicates of the above Western blot normalized to γ-tubulin. (TIF) [file pone.0050895.s008.tif]
