# Supplementary material for: MiR-27a Functions as a Tumor Suppressor in Acute Leukemia by Regulating 14-3-3θ
Source: PLoS One. 2012 Dec 7;7(12):e50895. doi: 10.1371/journal.pone.0050895 (PMC3517579; doi:10.1371/journal.pone.0050895)
Supplement: Table S1 — Primer sets used to clone predicted miR binding sites into the Luc reporter plasmid and to delete the seed region complement. (DOCX) [file pone.0050895.s009.docx]

**Table S1:** **Primer sets used to clone predicted miR binding sites into the Luc reporter plasmid and to delete the seed region complement**.

| **Luc primers** | **Left primer** | **Right primer** |
| --- | --- | --- |
| 14-3-3-θL-27a | 5’- CATG*GAATTC* GCCTTCCTGGT GTGCAGTA-3’ | 5’-CG*CTCGAG* GAAACCCCCGAAGAGT AAGG-3’ |
| 14-3-3βL-3’UTR | 5’-CATG*GAATTC* GCAACTTGTGCT TCAATAGTGG-3’ | 5’-CG*CTCGAG* ATTCACAGGTGCAACC ACAG-3’ |
| 14-3-3βL-1537 | 5’-CATG*GAATTC* CACAGTAATGCT GCCGTTGT-3’ | 5’-CG*CTCGAG* GGGGTTCAGTGTGGTG TGTT-3’ |
| 14-3-3βL-2061 | 5’-CATG*GAATTC* GCCGTGTGTTTT ATGAATGACCTTATC-3’ | 5’-CG*CTCGAG* GGAAGCAGAGGGACA GAAACTT-3’ |
| 14-3-3ζL-1266 | 5’- CATG*GAATTC* TGTGGCTTCAAA AGGGCCAGT-3’ | 5’-CG*CTCGAG* TCTGCAGCTGTAGTCA AAGGTGT-3’ |
| 14-3-3ζL-157 | 5’-CATG*GAATTC* GGACTACGACGT CCCTCAAA-3’ | 5’- CG*CTCGAG* TTGGATAATTCAGCT CCTTGC-3’ |
| 14-3-3ζL-627 | 5’-CATG*GAATTC* TGCCGCTGGTGA TGACAAGAA-3’ | 5’-CG*CTCGAG* AGCAATGGCTTCATC AAAAGCTGTC-3’ |
| 14-3-3ζL-1075 | 5’-CATG*GAATTC* CATTTGTCATCC ATGCTGTCCCACA-3’ | 5’-CG*CTCGAG* TCCCCGCCAGGACAAA CCAGTA-3’ |
| **QC primers** | **Left Primer** | **Right Primer** |
| 14-3-3θL-27a-del | 5’-GACTGTGTTTTGTATTAATAAG AAAATGTAG-3’ | 5’-CTACATTTTCTTATTAATACAAAA CACAGTC-3’ |
| 14-3-3βL-3’UTR-del1 | 5’-CTACTTTCATTGTTAACAAAGA AAAAAAGCCGTGTG-3’ | 5’- CACACGGCTTTTTTTCTTTGTTAAC AATGAAAGTAG-3’ |
| 14-3-3βL-3’UTR-del2 | 5’-CTAGTCCCAACTATTTGGGAGG GGAGGATCGCTTGAGCCCAGG-3’ | 5’- CCTGGGCTCAAGCGATCCTCCCCT CCCAAATAGTTGGGACTAG-3’ |
| 14-3-3βL-CDS-del1 | 5’-GCCAAACTCGCTGAGCAGGTAT GATGATATGGCTGCAGC-3’ | 5’- GCTGCAGCCATATCATCATACCTG CTCAGCGAGTTTGGC-3’ |
| 14-3-3βL-CDS-del2 | 5’- GCAGGTATGATGATATGGCTGA AGGCAGTCACAGAACAG-3’ | 5’- CTGTTCTGTGACTGCCTTCAGCCA TATCATCATACCTGC-3’ |
| 14-3-3βL-1537-del | 5’- CGTTGTTCGGGACTTAAAGACC TGTTTGGGCTGTTGCCAC-3’ | 5’- GTGGCAACAGCCCAAACAGGTCTT TAAGTCCCGAACAAC G-3’ |
| 14-3-3βL-2061-del | 5’- CAAAAAAAGGAATGATGTTAT GGCCTTTCACTTGAGGAG-3’ | 5’- CTCCTCAAGTGAAAGGCCATAACA TCATTCCTTTTTTTG-3’ |
|  |  |  |

Italicized bases indicate the restriction site used in cloning; GGATTC (EcoRI) and CTCGAG (XhoI). Underlined bases indicate bases added for optimal restriction enzyme cleavage.
