# Supplementary material for: MiR-27a Functions as a Tumor Suppressor in Acute Leukemia by Regulating 14-3-3θ
Source: PLoS One. 2012 Dec 7;7(12):e50895. doi: 10.1371/journal.pone.0050895 (PMC3517579; doi:10.1371/journal.pone.0050895)
Supplement: Table S2 — Correlation of GFP+ expression and pI+-staining cells in Molt16 FUGW/miR-27a-transduced cells. (DOCX) [file pone.0050895.s010.docx]

**Table S2: Correlation of GFP^+^ expression and pI^+^-staining cells in Molt16 FUGW/miR-27a-transduced cells.**

| %GFP+ cells | %pI+ cells |
| --- | --- |
| 94 | 92 |
| 73 | 69 |
| 89 | 86 |
| 82 | 77 |
| 45 | 40 |
| 61 | 56 |
| 65 | 63 |
| 33 | 28 |

Molt16 cells were FACS-analyzed for GFP expression and PI staining.
