# Supplementary material for: MiR-27a Functions as a Tumor Suppressor in Acute Leukemia by Regulating 14-3-3θ
Source: PLoS One. 2012 Dec 7;7(12):e50895. doi: 10.1371/journal.pone.0050895 (PMC3517579; doi:10.1371/journal.pone.0050895)
Supplement: Table S3 — Predicted binding sites for miR-23a cluster members in 14-3-3 isoforms. (DOCX) [file pone.0050895.s011.docx]

**Table S3: Predicted binding sites for miR-23a cluster members in 14-3-3 isoforms.**

| **14- 3-3-3 Isoform** | **miR-23a** | **miR-27a** | **miR-24** |
| --- | --- | --- | --- |
| 14-3-3θ (YWHAQ or 14-3-3τ) | none | **1177-1197** | none |
| 14-3-3β (YWHAB) | none | **1537-1557** | **321-342** |
|  |  | **2061-2081** | **343-364** |
|  |  |  | **1786-1807** |
|  |  |  | **1913-1934** |
|  |  |  | 2459-2480 |
| 14-3-3ζ (YWHAZ) | none | 724-744 | **157-178** |
|  |  | 803-823 | **171-192** |
|  |  | **1266-1286** | **627-648** |
|  |  |  | **1075-1096** |
|  |  |  | **1155-1176** |
| 14-3-3γ (YWHAG) | 2013-2033 | 313-333 | 304-325 |
|  | 2111-2131 | 487-507 | 611-632 |
|  |  | 1973-1993 | 3674-3695 |
|  |  | 2597-2617 |  |
|  |  | 2618-2638 |  |
| 14-3-3ε (YWHAE) | none | none | none |
| 14-3-3η (YWHAH) | 661-681 | none | none |
| 14-3-3σ (Stratifin) | none | none | none |

Predicted target sites for the miR-23a cluster were identified using TargstScan, PicTar, and RNA22 (19-22). Numbers refer to the 5’ to 3’ bp region in the mRNA sequence of interest. For RNA22, the following accession numbers were used for the 14-3-3 isoforms: NM_006826.2 (14-3-3-θ), NM_003404.3 (14-3-3β), NM_003406.3 (14-3-3ζ), NM_012479.3 (14-3-3γ), NM_006761.4 (14-3-3ε), NM_003405.3 (14-3-3η), and NM_006142.3 (14-3-3σ). Predicted sites in bold were cloned and analyzed in this study.
